# Unleashing the Potential of Defect Engineered Persistent Pr^3+^‐Activated Phosphors for Multi‐Dimensional Anti‐Counterfeiting and X‐Ray Imaging Applications

**DOI:** 10.1002/smll.202501752

**Published:** 2025-04-24

**Authors:** Annu Balhara, Santosh K. Gupta, Partha Sarathi Ghosh, Malini Abraham, Mohit Tyagi, Ashok Kumar Yadav, Subrata Das, Kathi Sudarshan, P. S. Sarkar

**Affiliations:** ^1^ Radiochemistry Division Bhabha Atomic Research Centre Trombay Mumbai 400085 India; ^2^ Homi Bhabha National Institute Anushaktinagar Mumbai 400094 India; ^3^ Glass & Advanced materials Division Bhabha Atomic Research Centre Trombay Mumbai 400085 India; ^4^ Materials Science and Technology Division CSIR‐National Institute for Interdisciplinary Science and Technology Thiruvananthapuram Kerala 695019 India; ^5^ Academy of Scientific and Innovative Research (AcSIR) Ghaziabad 201002 India; ^6^ Technical Physics Division Bhabha Atomic Research Centre Mumbai 400085 India; ^7^ Atomic & Molecular Physics Division Bhabha Atomic Research Centre Mumbai 400085 India

**Keywords:** anti‐counterfeiting, defect engineered phosphors, persistent luminescence, Pr^3+^ activation, X‐ray imaging

## Abstract

Persistent luminescence (PersL) in inorganic phosphors offers great potential for anti‐counterfeiting and optical storage, with optimization of PersL, multicolor tuning, and defect engineering. This study presents a Ca_3_Ga_2_Ge_3_O_12:_Pr^3+^ (CGGO:Pr) phosphor with long‐lasting PersL and multicolor emissions. Aliovalent codoping with Er^3+^ and Yb^3+^ ions optimizes deep/shallow trap redistribution, controlling trap depths from 0.98 to optimal 0.73 eV through the creation of new shallow electron traps (Yb_Ca_^•^, Er_Ca_^•^) alongside existing V_O_ levels. The smart Pr^3+^/Er^3+^/Yb^3+^:CGGO phosphor exhibits three‐dimensional visible emissions under 275  and 380 nm excitation, as well as upconversion emissions under 980 nm laser irradiation. Hybrid density functional calculations, thermoluminescence, and positron annihilation lifetime spectroscopy revealed the nature and density of different traps controlling the PersL. Together, a single system featuring multicolor luminescence has been developed, exhibiting improved PersL and regulated trap depths (≈0.73 eV) suitable for robust and multimodal anti‐counterfeiting of documents, pharmaceuticals, and industrial products. Furthermore, the composite PMMA‐CGGO:Pr phosphor films have shown remarkable capability for X‐ray imaging, achieving a resolution of 4 lp/mm, which exceeds that of commercial Gd_2_O_2_S:Tb screens. These findings highlight the potential of this work for advanced anti‐counterfeiting and X‐ray imaging applications, offering enhanced PersL with controlled trap depths.

## Graphical abstract

Enhanced multicolor persistent luminescence with optimized trap depths (≈0.73 eV) in Ca_3_Ga_2_Ge_3_O_12_:Pr^3+^ phosphors enable advanced anti‐counterfeiting, optical storage, and high‐resolution X‐ray imaging (4 lp mm^−1^), surpassing commercial screens.

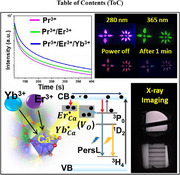

## 1 Introduction

Long persistent materials have continuously attracted great research interests in recent decades all thanks to their rapidly growing applications including anti‐counterfeiting, information storage, safety displays, photocatalysis, emergency lighting, bioimaging, and the health sector.^[^ ^1^, ^2^, ^3^, ^4^, ^5^, ^6^ ^]^ Persistent luminescence (PersL) is an interesting optical phenomenon that results in delayed emission from a material even after ceasing the excitation. The PersL in inorganic phosphors has been explored across different regions of the electromagnetic spectrum, including ultraviolet (UV), visible and near‐infrared (NIR) under ionizing radiation (e.g., X‐rays, α or γ rays), and UV or visible light as the excitation sources.^[^ ^2^ ^]^ Especially, the defect‐rich inorganic phosphor materials are widely investigated to attain prolonged PersL, as the excitation energy may be captured by numerous trap centers during the charging, and the stored energy contributes to the delayed emission.^[^ ^4^, ^7^ ^]^

The global market for anti‐counterfeiting technology has grown dramatically in recent years due to huge losses caused by the piracy of currency, documents, pharmaceuticals, and other industrial products.^[^ ^8^, ^9^ ^]^ The traditional single‐level anti‐counterfeit methods including the use of barcodes, seals, and holograms, can be easily replicated and are insufficient for high‐security anti‐counterfeiting. In the past decade, luminescent materials for anti‐counterfeiting have been reported widely in the form of invisible security inks or coding patterns that can be identified by distinct emissions under irradiation of light/laser light.^[^ ^10^, ^11^ ^]^ As a result, inorganic PersL materials that display multicolor luminescence when exposed to multi‐wavelength excitations are promising options for creating anti‐counterfeiting features with multiple encoding levels to combat advanced counterfeiting.^[^ ^12^, ^13^ ^]^

Lanthanide ions (Ln^3+^)‐doped phosphors are well‐known for their multicolor emissions from 4f‐4f transitions, covering a wide range from UV, visible to NIR under multi‐wavelength excitations from X‐rays, UV to NIR laser sources.^[^ ^14^, ^15^, ^16^, ^17^ ^]^ Especially, the unique blend of luminescence and upconversion (UC) emissions from Ln^3+^ ions can be utilized for designing inorganic phosphors that exhibit multimodal luminescence. The Ln^3+^‐doped inorganic hosts offer several advantages like excellent physicochemical stability, good mechanical properties, and tolerance to harsh conditions.^[^ ^18^, ^19^ ^]^ Crucially, the abundant inherent defects in inorganic phosphors resulting from high‐temperature synthesis and trap engineering through doping can be effectively harnessed to achieve long PersL.^[^ ^3^, ^5^, ^6^, ^9^, ^20^ ^]^ Moreover, various PersL phosphors based on Eu^3+^, Dy^3+^, Cr^3+^, Eu^2+^, Mn^2+^, Ce^3+^, Sm^3+^, Bi^3+^, Mn^4+^, and Pr^3+^, have been reported for multifunctional applications involving PersL.^[^ ^3^, ^21^, ^22^, ^23^, ^24^ ^]^ Among the various dopants, Pr^3+^‐activated inorganic phosphors have been reported to exhibit multiple emissions involving transitions between 4f5d and 4f levels, spanning a broad spectral range from UV and blue, to red and NIR, while also showcasing excellent afterglow properties.^[^ ^5^, ^25^, ^26^, ^27^, ^28^, ^29^, ^30^ ^]^ Importantly, the multicolor emissions of Pr^3+^ have been attained on X‐ray as well as UV to blue light excitations.^[^ ^31^, ^32^, ^33^, ^34^, ^35^, ^36^ ^]^ Chen et al.^[^ ^37^ ^]^ reported a red PersL in Pr^3+^‐doped YNbO_4_ phosphor for promising dynamic anti‐counterfeiting. Xiao et al.^[^ ^38^ ^]^ reported a Pr^3+^‐doped phosphor with multicolor emissions for anticounterfeiting technology. In addition, the Pr^3+^‐based PersL has been documented in numerous inorganic hosts such as Sr_2_Ga_2_GeO_7_,^[^ ^39^ ^]^ La_3_GaGe_5_O_16_,^[^ ^40^ ^]^ BaGa_2_O_4_,^[^ ^41^ ^]^ BaLu_2_Al_2_Ga_2_SiO_12_,^[^ ^42^ ^]^ Li_2_CaSiO_4_,^[^ ^43^ ^]^ Cd_3_Ga_2_Ge_3_O_12_,^[^ ^44^ ^]^ LiGa_5_O_8_,^[^ ^45^ ^]^ LiLuSiO_4_,^[^ ^46^ ^]^ Y_3_Al_2_Ga_3_O_12_,^[^ ^47^ ^]^ etc. Additionally, the high absorption of X‐ray excitation energy enables the use of Ln^3+^‐activated radioluminescent materials for potential X‐ray imaging and detection applications in medical diagnosis, security, and X‐ray information storage.^[^ ^13^, ^48^, ^49^, ^50^ ^]^ The X‐ray scintillating properties of Pr^3+^‐activated materials have been explored for radiation detection and imaging.^[^ ^51^, ^52^, ^53^ ^]^ More importantly, the X‐ray‐activated PersL allows the storage of optical information that can be employed for potential encryption applications in X‐ray imaging.^[^ ^13^, ^54^ ^]^

Concerning the expanding applications of long PersL, the development of inorganic phosphors with tunable PersL and desired trap levels is of utmost importance. Various approaches such as aliovalent doping, non‐stoichiometric synthesis, high‐temperature annealing, and lanthanide codoping have been effective in controlling the PersL.^[^ ^3^, ^20^ ^]^ Among these, the aliovalent lanthanide codoping strategy is effective for designing PersL phosphors intended for multimodal anti‐counterfeiting, as the combination of codopants offer spectral multiplexing while also introducing intermediate trap levels for controlled PersL. The simultaneous codoping of Ln^3+^ ions in PersL inorganic phosphors facilitates multicolor emission, afterglow, and upconversion luminescence (UCL) when stimulated by various excitations, which can be used for multilevel anti‐counterfeiting applications. For instance, long PersL phosphors with UCL, such as NaLa(MoO_4_)_2_:Pr^3+^/Yb^3+^,^[^ ^55^ ^]^ CaTiO_3_:Pr^3+^/Er^3+^,^[^ ^56^ ^]^ and (Ca, Zn)TiO_3_:Pr^3+^/Er^3+[^ ^57^ ^]^ have been explored recently. Huang et al.^[^ ^58^ ^]^ reported an effective approach of codoping rare‐earth ions in LiLuGeO_4_:Bi^3+^,Ln^3+^ (Ln = Pr, Tb, or Dy) phosphor to generate extra trap centers for potential anti‐counterfeiting applications, and information storage using X‐ray/UV charging. The codoping of Ln^3+^ presents a pioneering approach for enhancing afterglow, managing trap depths, and influencing the nature of defects, which aids in the development of novel prolonged PersL phosphors for advanced applications.

To achieve the multimodal emissions in a single material with strong PersL, codoping strategy of Pr^3+^ and Er^3+^ ions with distinct absorption and emission properties is used and the efficient Ca_3_Ga_2_Ge_3_O_12_ (CGGO) garnet phosphor was selected due to the suitable incorporation sites for Ln^3+^ ions. The Pr^3+^ and Er^3+^ ions displayed strong multimodal emissions under different excitation wavelengths (275 and 380 nm) ranging from visible to NIR spectral ranges. The synchrotron‐based advanced techniques such as extended X‐ray absorption fine structure (EXAFS) and X‐ray absorption near edge structure (XANES) were employed to study the local structure, occupation sites, and oxidation states of dopants in CGGO host. In addition, density functional theory (DFT) calculations and positron annihilation lifetime spectroscopy (PALS) studies were performed to understand the intrinsic and defect creation on codoping in CGGO matrix and identify the mechanism of strong PersL of Pr^3+^ ions. The PersL and thermoluminescence (TL) studies were carried out to understand the trap levels and codoping effect on PersL of Pr^3+^ ions. Moreover, the trap density was increased significantly and trap depth were tuned to optimum range via aliovalent Er^3+^/Yb^3+^ codoping approach. Further, the potential of composite PMMA and Pr^3+^‐doped CCGO (CGGO:Pr) phosphor films is demonstrated for X‐ray imaging. Additionally, the smart tridoped‐Pr^3+^/Er^3+^/Yb^3+^:CGGO (CGGO:Pr/Er/Yb) phosphor was rationally designed to show three‐dimensional visible emissions under distinct excitation wavelengths at 275 and 380 nm along with the UC emissions under 980 nm laser irradiation. All the codoped samples exhibit strong afterglow emission of Pr^3+^ ions after ceasing the UV light excitation. The synergistic effect of Ln^3+^ codoping strategy in CGGO:Pr phosphors help in realizing the multimodal luminescence which is promising for robust and high‐level anticounterfeiting coding. The visually observed PersL is the added advantage to design complex anti‐counterfeiting coding for documents and industrial entities that must be difficult to replicate.

## 2 Results and Discussion

### 2.1 Phase and Structural Analysis

**Figure 1:**
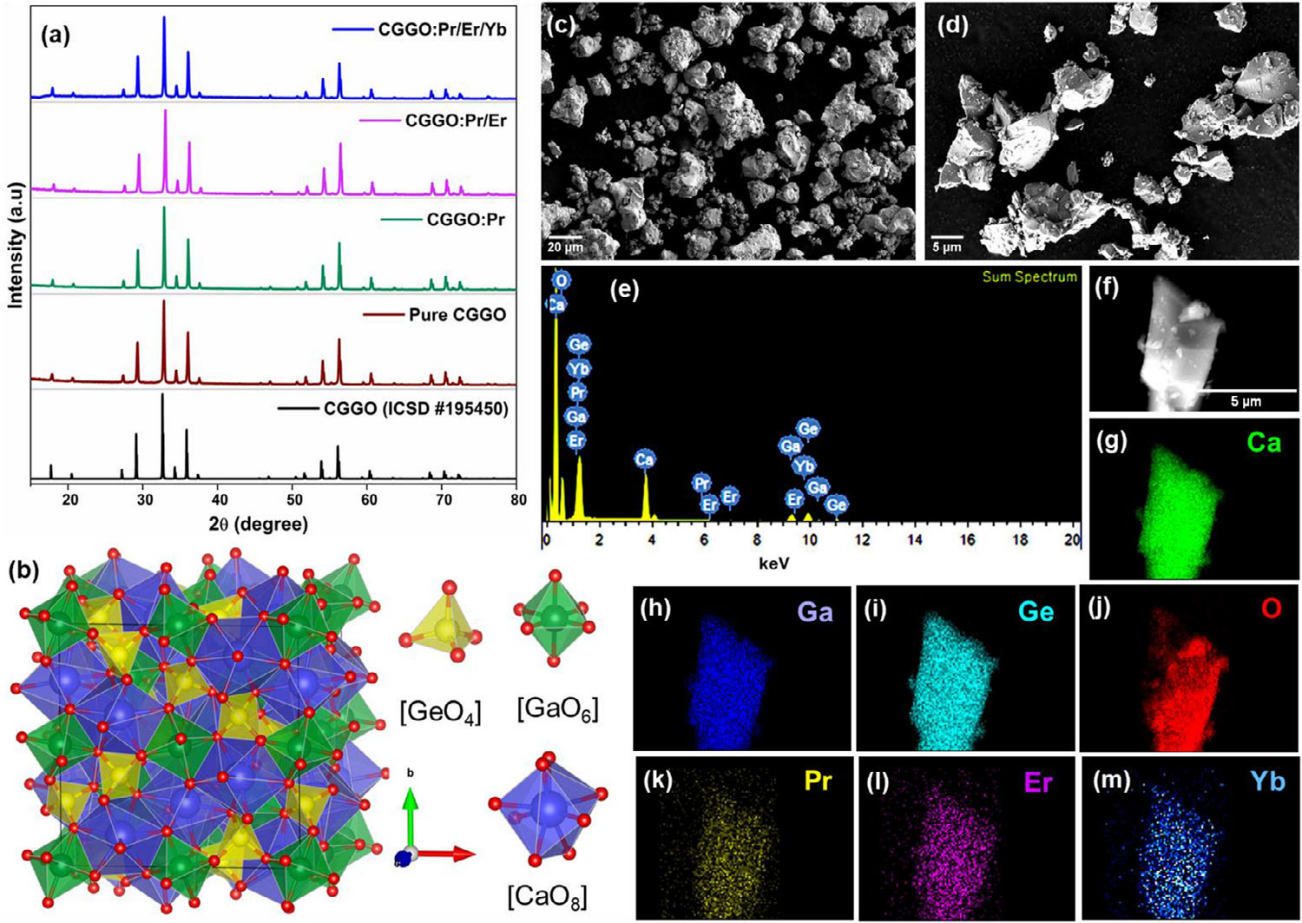
a) XRD patterns of pure CGGO, CGGO:Pr, CGGO:Pr/Er, and CGGO:Pr/Er/Yb phosphors. b) Crystal structure of Ca_3_Ga_2_Ge_3_O_12_. c,d) FE‐SEM images of CGGO:Pr/Er/Yb sample. e) EDS spectra of CGGO:Pr/Er/Yb sample. f–m) Elemental mapping of CGGO:Pr/Er/Yb sample.

**Figure** **1**a displays the XRD patterns of pure CGGO, CGGO:0.03Pr (CGGO:Pr), CGGO:0.03Pr/0.03Er (CGGO:Pr/Er), and CGGO:0.03Pr/0.03Er/0.02Yb (CGGO:Pr/Er/Yb) samples. The XRD peaks observed in the pure and doped CGGO samples have good agreement with the standard (ICSD No. 195 450) which confirmed the formation of pure crystalline CGGO phase in all the samples. CGGO belongs to the cubic phase (space group Ia‐3d) and the crystal structure is represented by Figure 1b. The Ca^2+^ ions occupy dodecahedron sites (CaO_8_), Ga^3+^ is located as GaO_6_ octahedrons, and Ge^4+^ ions is present in tetrahedral sites (GeO_4_). The Pr^3+^ ions are more likely to be introduced at Ca^2+^ sites (CN = 8, Ca^2+^ = 1.12 Å and Pr^3+^ = 1.126 Å) compared to the Ga^3+^ octahedral sites (CN = 6, Ga^3+^ = 0.62 Å and Pr^3+^ = 0.99 Å) because of the similar ionic radii. The EXAFS analysis and DFT calculations were performed to confirm the site occupation of Pr^3+^ ions in CGGO (discussed in the next section).

The field emission scanning electron microscopy (FE‐SEM) images of tri‐doped CGGO:Pr/Er/Yb sample show the formation of agglomerated micron size grains (Figure 1c,d). The energy dispersive X‐ray spectroscopy (EDS) was performed on CGGO:Pr/Er/Yb sample and the EDS spectra confirmed the presence of Ca, Ga, Ge, O, Pr, Er, and Yb elements (Figure 1e). The elemental mapping of different elements in CGGO:Pr/Er/Yb sample shown in Figure 1f–m indicated the uniform distribution of Pr^3+^, Er^3+^ and Yb^3+^ ions. The Fourier‐transform infrared (FTIR) spectroscopy was performed on pure CGGO and all the codoped CGGO samples (Figure S1, Supporting Information). The bands observed in the 600 to 800 cm^−1^ can be assigned to characteristic GaO_6_ and GeO_4_ stretching vibrations in CGGO host. Figure S2 (Supporting Information) presents the X‐ray photoelectron spectroscopy (XPS) results of CGGO:Pr sample and the XPS survey spectra confirmed the presence of Ca, Ga, Ge, O, and Pr. The peaks with binding energies of 346.77/350.21, 1118.35/1145.41, and 32.78 can be assigned to the Ca 2p, Ga 2p, and Ge 3d, respectively. The O 1s XPS spectrum was deconvoluted into two peaks at 530.34 and 531.62 eV, which were ascribed to lattice oxygen and oxygen vacancies. The Pr 3d peak in the XPS profile was observed at the binding energy of 932.88 eV which indicated the presence of Pr ion in the trivalent state.^[^ ^59^ ^]^

### 2.2 XANES and EXAFS Measurements

**Figure 2:**
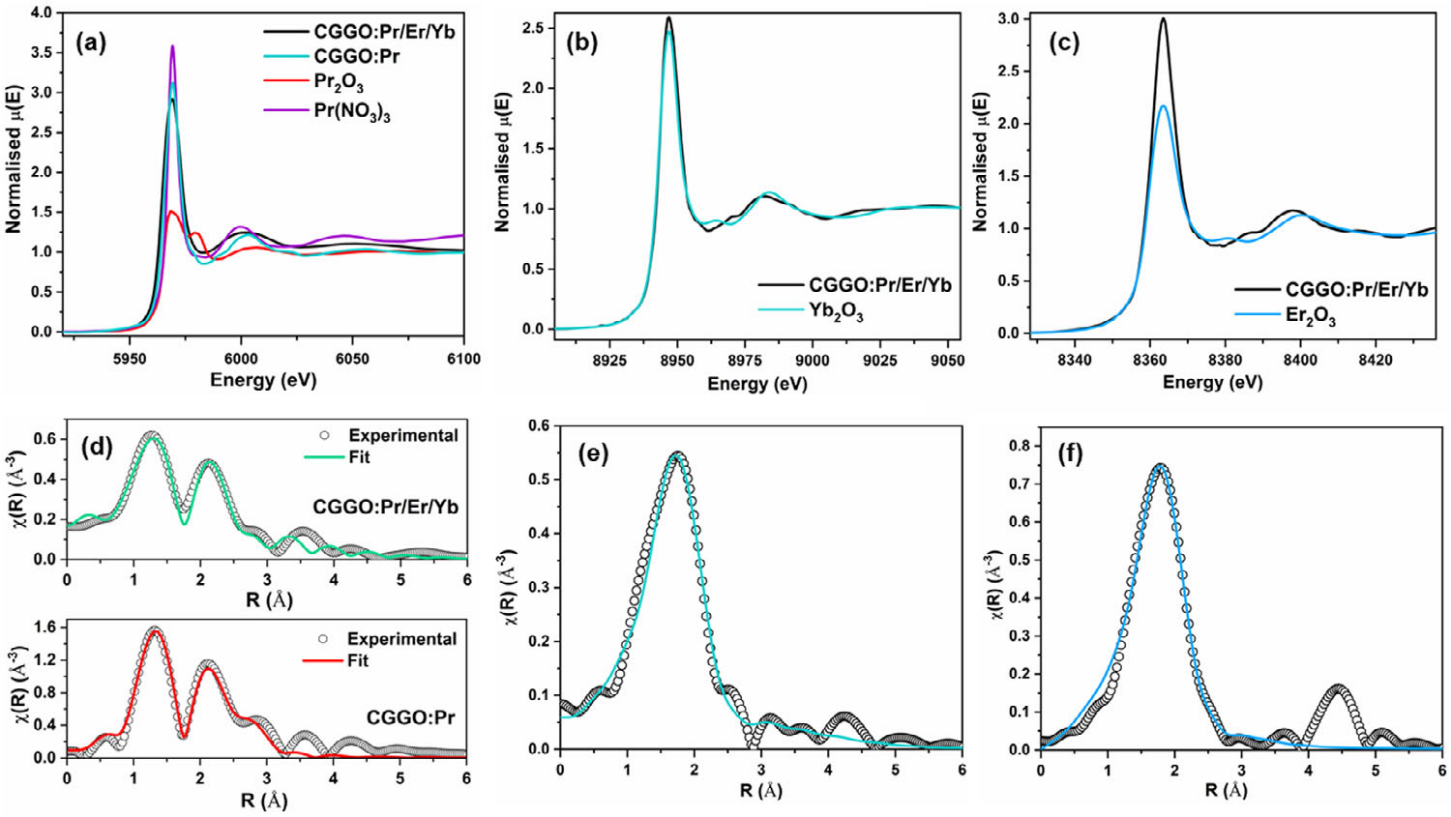
a) Normalized XANES spectra of CGGO:Pr and CGGO:Pr/Er/Yb at Pr L3‐edge along with Pr_2_O_3_ and Pr(NO_3_)_3_ standard. Normalized XANES spectrum of CGGO:Pr/Er/Yb at b) Yb L3‐edge along with Yb_2_O_3_ standard and c) Er L3‐edge along with Er_2_O_3_ standard. Fourier transformed EXAFS spectra (scatter points) and its respective fittings (solid line) of d) CGGO:Pr and CGGO:Pr/Er/Yb at Pr L3‐edge, e) CGGO:Pr/Er/Yb at Yb L3‐edge, and f) CGGO:Pr/Er/Yb at Er L3‐edge.

The normalised XANES spectrum at Pr L3‐edge is shown for CGGO:Pr along with Pr_2_O_3_ standard. The peak position of the white line (5968 eV) in the Pr L3‐edge XANES spectrum of Pr doped CGGO is identical to those of Pr_2_O_3_ (**Figure** **2**a). This indicates that the Pr species that the Pr species in CGGO:Pr and CGGO:Pr/Er/Yb share the same average +3 oxidation state, however different whiteline peak intensity may also indicate change in oxidation state. It should also be noted that the coordination geometry can influence the intensity of the whiteline peak, even for the same oxidation state. Hence, an additional XANES measurements have been performed for another +3 oxidation state standard, i.e., Pr(NO_3_)_3_, to provide a more comprehensive comparison. It was observed that the whiteline peak position and intensity of both the CGGO:Pr and CGGO:Pr/Er/Yb samples clearly align with the characteristics typically associated with the Pr^3+^ ion. This comparison to the Pr(NO_3_)_3_ standard, a well‐known reference for Pr^3+^, further confirms the trivalent oxidation state of Pr in our sample.

Also, the strong whiteline peak intensity of CGGO:Pr indicating changes in the coordination environment that may be due to increased coordination number or change in occupation site. A denser local electronic structure around the absorber tends to enhance the peak intensity.

The normalized XANES spectrum of CGGO:Pr/Er/Yb at the Yb L3‐edge is shown in Figure 2b, along with that of standard Yb_2_O_3_. The coincidence of the white‐line peak between Yb_2_O_3_ and the sample confirms the +3‐oxidation state of Yb in the doped material. However, the differences in the post‐edge oscillations suggest a distinct coordination environment for Yb in the sample compared to standard. The normalized XANES spectrum of CGGO:Pr/Er/Yb at the Er L3‐edge is shown in Figure 2c, alongside the spectrum of standard Er_2_O_3_. The alignment of the white‐line peak between Er_2_O_3_ and the sample confirms the +3‐oxidation state of Er in the doped material. However, the stronger white‐line peak intensity observed for Er‐doped CGGO suggests changes in the coordination environment, such as an increased coordination number or a variation in ligand type, similar to what was observed in the case of Pr doping.

The local structure was determined through the EXAFS analysis of the spectrum. The XAS analysis has been carried out using the Demeter package, which incorporates ATHENA and ARTEMIS subroutines for data processing and analysis.^[^ ^60^ ^]^ The theoretical EXAFS spectra were simulated using the structural parameters acquired from Rietveld‐refined XRD structures. The bond length and disorder factors (σ^2^) were varied for the EXAFS analysis. Fourier transform spectra presented here are phase‐uncorrected, resulting in the coordination peak appearing at slightly lower inter‐atomic distances compared to the actual bond length. Figure 2d presents the Fourier transform spectrum of CGGO:Pr and CGGO:Pr/Er/Yb samples at the Pr L3‐edge, along with the best‐fitting curve. Two prominent peaks are observed at 1.25 and 2.05 Å. The Fourier transform EXAFS spectra suggested that the coordination peaks of Pr^3+^ in singly doped CGGO:Pr sample have good similarity with the co‐doped CGGO:Pr/Er/Yb sample. The fitting results in CGGO:Pr shows Pr‐O coordination at a distance of 1.98 ± 0.02 Å. The first peak corresponds to Pr^3+^ substituting Ga^3+^ at the six‐coordinated Ga^3+^ site, with an interatomic distance of 1.96 ± 0.02 Å which is comparable to the CGGO:Pr sample. The second peak arises from Pr^3+^ substituting Ca^2+^ at the eight‐coordinated Ca^2+^ site, with an interatomic average distance of 2.40 ± 0.03 Å. Mixed‐phase fitting analysis indicates a contribution of 45 ± 5% from the Ga site, with the remainder attributed to the Ca^2+^ site, suggesting an almost equal distribution of Pr^3+^ across both sites.

Figure 2e shows the Fourier transform EXAFS spectrum of CGGO:Pr/Er/Yb at the Yb L3‐edge. The spectrum exhibits a strong and broad first coordination peak ≈1.75 Å. The substitutional model, in which Ca^2+^ is replaced by Yb^3+^, successfully fits the spectrum. The fitting results indicate the contribution of two Yb‐O coordination shells to this coordination peak, with four oxygen atoms in each shell at distances of 2.21 ± 0.02 and 2.33 ± 0.03 Å, respectively. This clearly confirms the substitution of Yb^3+^ exclusively at Ca^2+^ sites. The Fourier transform EXAFS spectrum of CGGO:Pr/Er/Yb at the Er L3‐edge shows a strong coordination peak ≈1.77 Å (Figure 2f). The fitting results indicate contributions from two Er‐O coordination shells, each with a coordination number of four, at distances of 2.25 ± 0.01 and 2.37 ± 0.02 Å, respectively. The best‐fitting curve, shown in Figure 2f alongside the experimental spectrum, confirms these results. These bond lengths suggest the substitution of Er^3+^ at the Ca^2+^ site.

### 2.3 DFT Calculations

The crystal structure of CGGO garnet compound belongs to the body centered cubic system with the space group of Ia‐3d and the unit cell contains eight CGGO formula units with a total of 120 ions per unit cell.^[^ ^61^ ^]^ The Ca^2+^, Ga^3+^, and Ge^4+^ cations are surrounded by eight, six, and four oxygen anions forming a dodecahedron, an octahedron, and a tetrahedron, respectively. The DFT calculated lattice parameters of a = 12.408 Å match preferably with the experimentally determined value of a = 12.252 Å. The density of states (DOS) of pristine CGGO shows that the bottom of the valence band (VB) is formed by Ge‐d states, which are not dominant in the chemical bonding. Strong hybridization of O 2s states with 4p states of Ga and Ge is evident. The middle of the VB has been demonstrated to be occupied by d states of Ga, while Ga and Ge 4р states with a considerable admixture of O 2p states form the top of the VB. The Heyd, Scuseria, and Ernzerhof (HSE) hybrid functional calculated electronic bandgap of 5.2 eV is in agreement with experimentally determined bandgap of 5.5 eV.^[^ ^62^ ^]^

In order to decide the preferential occupation of Pr atom in CGGO, the cohesive energy of E(Ge_24_Ga_16_[Ca_21_Pr_2_V_ca_]O_96_), E(Ge_24_[Ga_14_Pr_2_]Ca_24_O_96_), and E(Ge_48_[Ga_30_Pr_2_][Ca_45_Pr_2_V_Ca_]O_192_) is calculated. The E(Ge_24_Ga_16_[Ca_21_Pr_2_V_ca_]O_96_) is the cohesive energy of Ge_24_Ga_16_Ca_24_O_96_ unit cell with 2Pr^3+^ atom doping at 2Ca^2+^ site and with a Ca^2+^ vacancy (V_Ca_^2+^). The E(Ge_24_[Ga_14_Pr_2_]Ca_24_O_96_) is the cohesive energy of Ge_24_Ga_16_Ca_24_O_96_ unit cell with 2Pr^3+^ atom doping at 2Ga^3+^ site. The E(Ge_48_[Ga_30_Pr_2_][Ca_45_Pr_2_V_Ca_]O_192_) is the cohesive energy of 1×1×2 supercell with 2Pr^3+^ doping at both Ca and Ga sites, and one V_Ca_^2+^ remains at Ca^2+^ site. In this formalism, a charge compensating mechanism: 3Ca^2+^ → 2Pr^3+^ + V_Ca_ is taken into consideration. The calculated energy difference calculated using Equation (1) was found to be −0.15 eV.

(1) $$\begin{array}{c} \Delta E=E\left({\mathrm{Ge}}_{48}\left[{\mathrm{Ga}}_{30}{\mathrm{Pr}}_{2}\right]\left[{\mathrm{Ca}}_{45}{\mathrm{Pr}}_{2}V_{\mathrm{Ca}}\right]O_{192}\right)-\\ E\left({\mathrm{Ge}}_{24}{\mathrm{Ga}}_{16}\left[{\mathrm{Ca}}_{21}{\mathrm{Pr}}_{2}V_{\mathrm{Ca}}\right]O_{96}\right)-\\ E\left({\mathrm{Ge}}_{24}\left[{\mathrm{Ga}}_{14}{\mathrm{Pr}}_{2}\right]{\mathrm{Ca}}_{24}O_{96}\right) \end{array}$$

This indicates the preferential occupation of Pr dopant simultaneously at Ca and Ga site rather than occupying at Ca and Ga site, separately. This result is in well correlation with the EXAFS results revealing two‐site occupation by Pr^3+^ ions in CGGO. A comparison of average polyhedral properties of GaO_6_/PrO_6_ and CaO_8_/PrO_8_ when Pr^3+^ is doped at Ga^3+^ and Ca^2+^ site simultaneously is shown in the Table S1 (Supporting Information). It is evident that the difference in CaO_8_/PrO_8_ polyhedral is minimal and distortion in the PrO_8_ polyhedra is also expected to be minimal. Local symmetry analysis also indicated that overall symmetry of the PrO_8_ polyhedra remain intact to CaO_8_ polyhedra. Contrarily, there is increase in average bond distances and polyhedral volumes due to Pr doping at Ga site and structural distortion is also high. Local symmetry analysis also indicated that overall symmetry of the PrO_6_ polyhedra is reduced compared to that of GaO_6_. Hence, the occupation of the dopant (in this case Pr^3+^) is decided not only by charge balancing mechanism but also by the local structural distortion.

**Figure 3:**
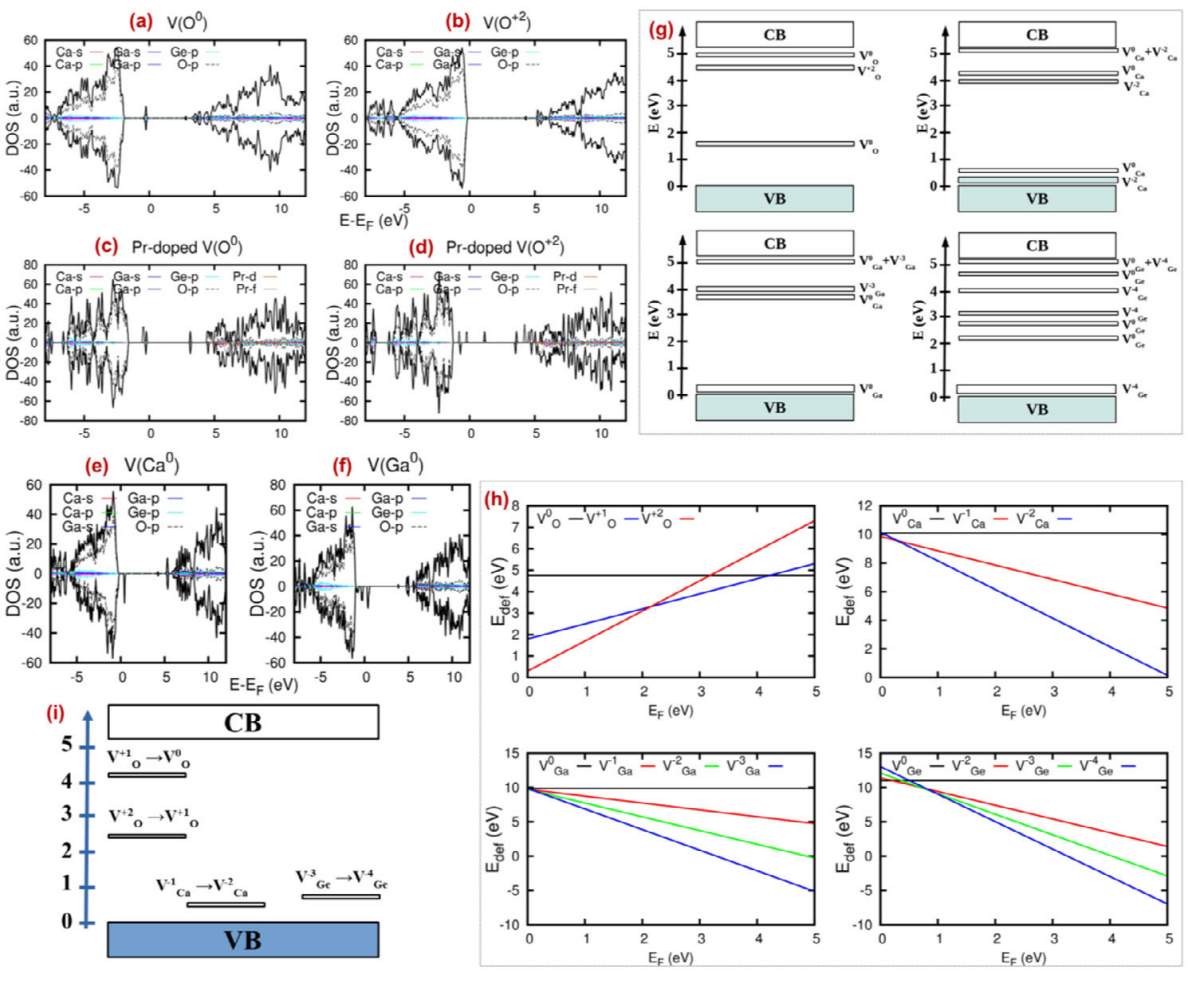
a) Total and angular momentum decomposed DOS of CGGO with a neutral oxygen vacancy (V_O_^0^) calculated using DFT‐HSE06, (b) DOS for CGGO with an oxygen vacancy of charge +2 (V_O_^+2^), c) DOS of Pr‐doped CGGO with a neutral oxygen vacancy (V_O_^0^), d) DOS of Pr‐doped CGGO with an oxygen vacancy of charge +2 (V_O_^+2^), e) DOS due to the presence of a neutral calcium vacancy (V_Ca_^0^), f) DOS due to the presence of a neutral gallium vacancy (V_Ga_^0^), g) overall summary of the defect state locations arising from different vacancies: V_O_^0^, V_O_^+1^, V_O_^+2^ (oxygen vacancies), V_Ca_^0^ and V_Ca_^−2^ (calcium vacancies), V_Ga_^0^ and V_Ga_^−3^ (gallium vacancies), and V_Ge_^0^ and V_Ge_^−4^ (germanium vacancies), h) DFT‐HSE06 calculated Fermi level (E_f_) as a function of the Fermi energy (EF) for charged defect states of oxygen, calcium, gallium, and germanium vacancies and i) Charge‐state transition levels with respect to the hydrogen reference energy (HRBE).

**Figure** **3**a–d shows DFT calculated total and angular momentum‐decomposed DOS due to the presence of a neutral O vacancy and O vacancy with a charge of 2+ (V_O_^+2^) in pure and Pr‐doped CGGO system. Similarly, Figure 3e,f shows the DFT calculated total and angular momentum‐decomposed DOS due to the presence of a neutral Ca vacancy (V_Ca_), neutral Ga vacancy (V_Ga_), neutral Ge vacancy (V_Ge_) (Figure S3, Supporting Information), Ca vacancy with a charge of 2+ (V_Ca_^+2^), Ga vacancy with a charge of 3+ (V_Ga_^+3^) and Ge vacancy with a charge of 4+ (V_Ge_^+4^) (Figure S4a–c, Supporting Information). The spin‐up and spin‐down components are shown separately in the upper and lower panels, respectively. The overall nature of the VB remains unaltered but “shallow” and “deep” impurity states appear above the VB maximum in the bandgap and below the Fermi level. This impurity bands are mainly composed of the O‐p states. Figure 3g shows a summary of various defect states that arises within the bandgap.

Understanding the defect formation energy is important as the photoluminescence mechanism is highly related to the charge state of the impurity dopant/defect. So, the formation energy of a defect X in a charge state q is defined as:

(2) $$E_{f}\left(X_{q}\right)=E_{\mathrm{tot}}\left(X_{q}\right)-E_{\mathrm{tot}}\left(\mathrm{bulk}\right)-n_{i}\mu_{i}+q\left(E_{F}+E_{\mathrm{VBM}}\right)+\mathrm{dE}$$

Here, E_tot_(X_q_) is the total energy derived from the supercell calculation with a defect X of charge q. The E_tot_(bulk) value corresponds to the pure system using an equivalent supercell. The integer n_i_ is corresponding to the number of atoms of type i that is added to (n_i_ > 0) or removed (n_i_ < 0) from the supercell for the formation of the defect system. The ‘µ_i_’ corresponds to the chemical potentials corresponding to the added or removed species, representing the energy of the reservoirs with which atoms are being exchanged. While considering the insulators, the defects can have various charge states, by the exchange of electrons within an electron reservoir. The corresponding energy is the electron chemical potential or Fermi level. The dE is a correction used to mitigate the interactions of the charges of defects with their periodic images. Here, we employ a Madelung correction as proposed by Leslie and Gillan.^[^ ^63^ ^]^

A neutral defect does not create electronic carriers. A positively‐charged defect has a positive slope in a defect diagram and represents a donor‐like defect for charged oxygen defect. In other words, the defect ionizes to a positively‐charged state by giving up electron(s) and creates donors like states as shown in Figure 3h. The variation of DFT calculated defect formation energies of O, Ca, Ga, and Ge in various charge states in the dilute limit as a function of Fermi energy is shown in Figure 3h (left panel). From the DFT calculated formation energies for different vacancies, it can be said that in comparison to V_O_^+1^ and V_O_^0^, the formation energies V_O_^+2^ defects are low in the 0 to 2.4 eV range (in E_F_–E_V_ scale) near the VB. Hence, oxygen vacancies may behave as n‐type defect to donate electrons. Similarly, we can say that V_O_^+1^ defects are favorably formed in the 2.4 to 4.2 eV (in E_F_–E_V_ scale) and beyond this range V_O_^0^ defects are favored to be formed. In case of Ca, Ga, and Ge charged vacancy states the slopes of the charge defect formation lines are negative and expected to give rise to acceptor like states. In case of Ca defect, the formation energy of V_Ca_^−1^ is lowest upto 0.4 eV and then V_Ca_^−2^ is most stable after that. In case of Ga, V_Ga_^−3^ is the most energetically favorable in whole E_F_–E_V_ range. Finally, in case of Ge, the formation energy of V_Ge_^−3 ^ is lowest upto 0.7 eV and then V_Ge_^−4 ^ is most stable after that.

Four charge‐state transition levels as observed from the present DFT calculation are shown in Figure 3h (right panel). Two of them are deep donor levels ε (+2/+1) at 2.4 eV (V_O_^+2^ → V_O_^+1^) and ε (+1/0) at 4.2 eV (V_O_^+1^ → V_O_^0^). We could find two charge transition states ε (‐1/‐2) and ε (‐3/‐4) close to VBM ≈0.4 and 0.7 eV, respectively, for (V_Ca_^−1^ → V_Ca_^−2^) and (V_Ge_^−4 ^ → V_Ge_^−4 ^). Here, we have defined the impurity levels as the charge‐state transition levels (ε (q_1_/q_2_), from charge q_1_ to q_2_) and we have considered the Fermi level locations where different defect states with different charges have the same formation energy. These charge‐state transition levels with respect to the host referred binding energies (HRBE) are shown in a conventional diagram on the right side of Figure 3h. These charge‐state transition levels with respect to the HRBE are shown in a conventional diagram in Figure 3i. The different defect level diagram show that the oxygen vacancies act as electron trap centers and cation vacancies as hole trap centers.

**Table** **1** shows that oxygen vacancy formation energy is lowest among all possible vacancy defects both in pristine and doped cell where Pr‐doping in done at both Ca and Ga sites. This implies formation of oxygen defects is most energetically favorable. Moreover, oxygen defect formation energies become lower with Pr doping signifies oxygen defect formation is even more preferable with Pr doping. As a result, the defect concentrations are expected to be higher in Pr doped CGGO.

**Table 1:** Defect formation energies of O, Ca, Ga, and Ge in neutral and charge states.

Defect type	Defect formation energy [eV]	
	Pristine	Pr‐doping in both Ca and Ga site
V_O_^0^ V_O_^+1^ V_O_^+2^	4.7 1.8 0.3	4.3 1.4 0.1
V_Ca_^0^	10.1	10.5
V_Ga_^0^	9.8	9.2
V_Ge_^0^	11.0	11.8

### 2.4 Photoluminescence Study

**Figure 4:**
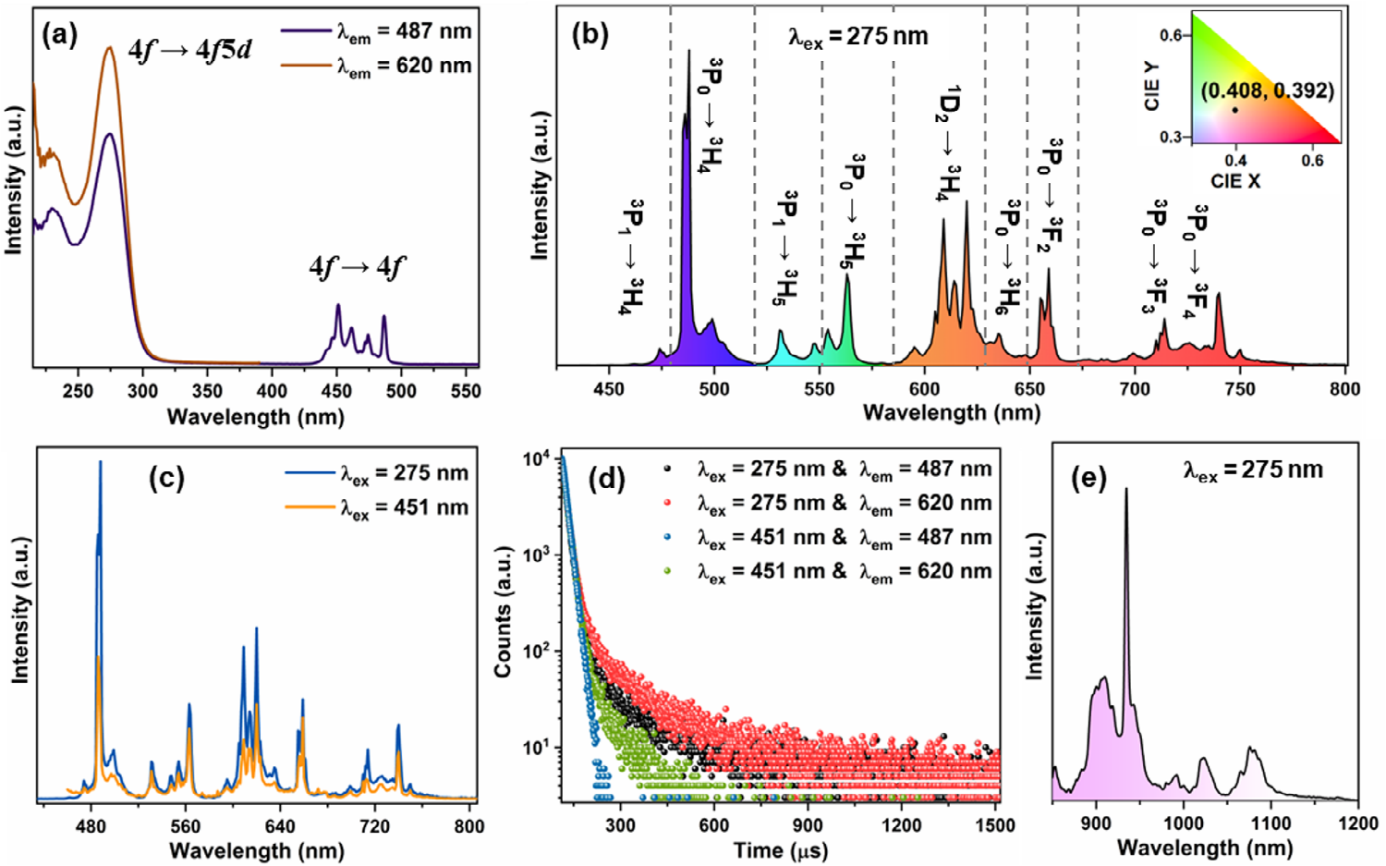
a) PLE spectra of CGGO:Pr monitored at emission wavelengths of 487 and 620 nm. b) Emission spectra of CGGO:Pr (λ_ex_ = 275 nm) (inset shows the CIE color coordinates). c) Emission spectra at λ_ex_ = 275 and 451 nm. d) Decay curves are monitored at different emission and under excitation wavelengths. e) NIR Emission spectra of CGGO:Pr (λ_ex_ = 275 nm).

The photoluminescence excitation (PLE) spectra of CGGO:Pr shows different absorption bands in UV and blue region that can be ascribed to 4f ⟶ 4f5d and 4f ⟶ 4f transitions of Pr^3+^ ions (see **Figure** **4**a). With 275 nm excitation, the emission spectra (Figure 4b) display the characteristic sharp emission bands of Pr^3+^ ions spanning from visible to NIR region (450–780 nm). The emissions bands peaked at 474, 487, 531, 563, 620, 635, 659, 713, and 740 nm can be assigned to the ^3^P_1_ → ^3^H_4_, ^3^P_0_ → ^3^H_4_, ^3^P_1_ → ^3^H_5_, ^3^P_0_ → ^3^H_5_, ^1^D_2_ → ^3^H_4_, ^3^P_0_ → ^3^H_6_, ^3^P_0_ → ^3^F_2_, ^3^P_0_ → ^3^F_3_, and ^3^P_0_ → ^3^F_4_ transitions, respectively.^[^ ^36^ ^]^ The inset of Figure 4b shows the CIE diagram of Pr^3+^ emission with color coordinates of (0.408, 0.392). The emission spectra of Pr^3+^ show similar spectral features under different excitation at 275 and 451 nm (Figure 4c). The decay curves for ^3^P_0_ → ^3^H_4_ and ^1^D_2_ → ^3^H_4_ transitions in CGGO:Pr were recorded with excitation at 275 and 451 nm (Figure 4d), and were best fitted in three exponential function as follows:

(3) $$I\left(t\right)=A_{1}\exp\left(-t/\tau_{1}\right)+A_{2}\exp\left(-t/\tau_{2}\right)+A_{3}\exp\left(-t/\tau_{3}\right)$$

Here, “I(t)” represents the luminescence intensity at “t” time, τ_1, _τ_2 _and τ_3_ are fitted lifetime components, and A_1_, A_2,_ and A_3_ denote pre‐exponential constants. The decay curves of Pr^3+^ luminescence deviate from single‐exponential decay function which can be ascribed to dual‐site occupation of Pr^3+^ ions at Ca^2+^/Ga^3+^ sites, and the presence of multiple de‐excitation routes such as radiative transition and multiphonon relaxation processes.^[^ ^64^ ^]^ The decay studies revealed that the ^1^D_2_ state (620 nm) has a longer lifetime of 90.9 µs than the ^3^P_0_ state (487 nm) having the lifetime of 56.8 µs which is due to spin‐allowed ^3^P_0_ → ^3^H_4_ transition. The ^1^D_2_ level decays slowly due to spin‐forbidden nature of ^1^D_2_ → ^3^H_4_ transition.^[^ ^65^ ^]^ The NIR emission spectra of Pr^3+^ ions show the bands at 908, 934, 1023, 1065, and 1080 nm which can be ascribed to the ^1^D_2_ → ^3^F_2_, ^1^D_2_ → ^3^F_3_, and ^1^D_2_ → ^3^F_4_ transitions (Figure 4e), respectively.^[^ ^45^ ^]^ The temperature‐dependent emission spectra of CGGO:Pr sample under 275 nm excitation show different intensity of 4f ⟶ 4f transitions with increased anti‐stokes emission from ^3^P_0_ and ^1^D_2_ states (Figure S5, Supporting Information).

**Figure 5:**
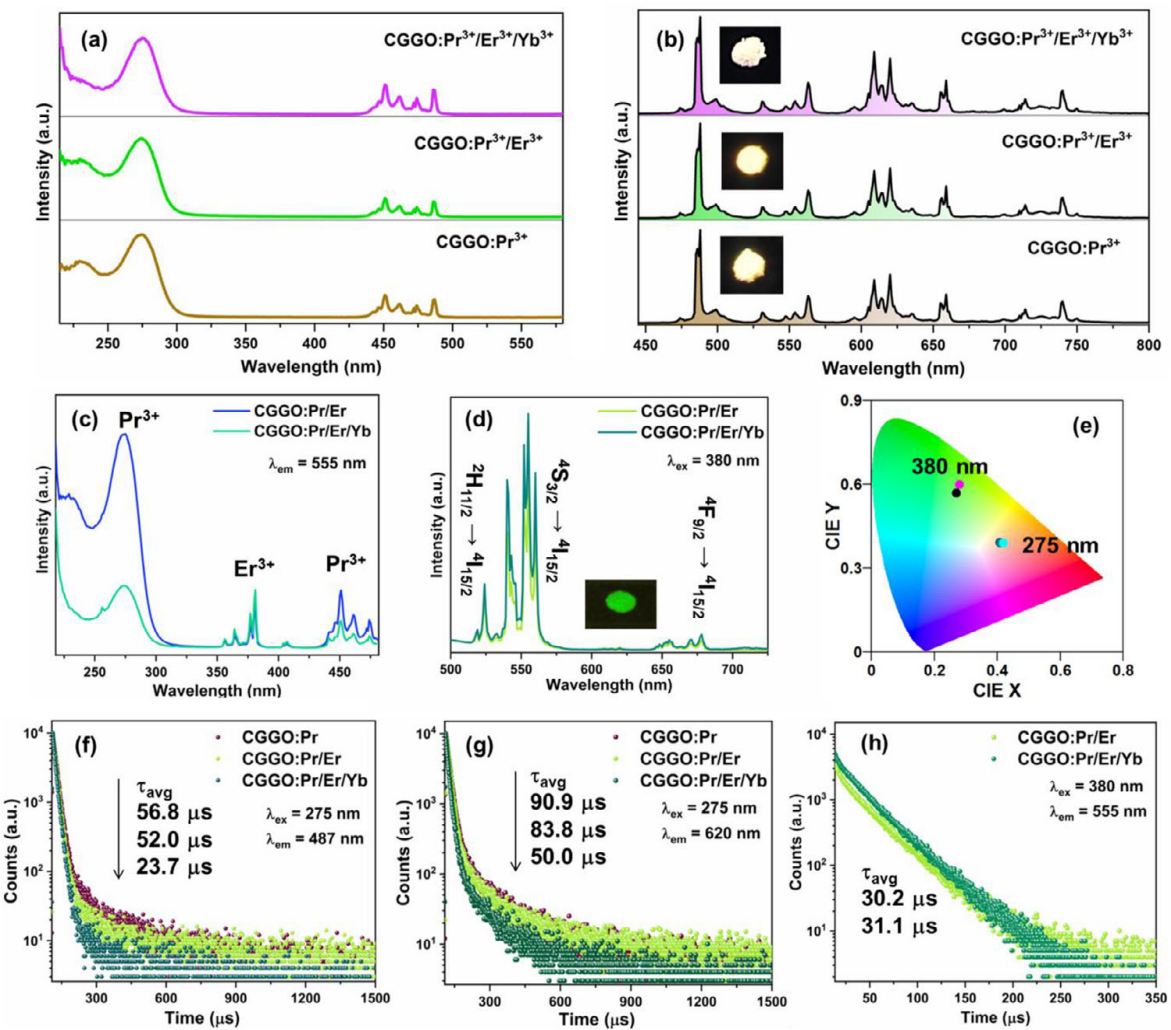
a,b) PLE (λ_em_ = 620 nm) and emission spectra (λ_ex_ = 275 nm) of Pr^3+^ ions in CGGO:Pr, CGGO:Pr/Er, and CGGO:Pr/Er/Yb phosphors, respectively. c,d) PLE (λ_em_ = 555 nm) and emission spectra of Er^3+^ in CGGO:Pr/Er and CGGO:Pr/Er/Yb (λ_ex_ = 275 nm) phosphors, respectively. e) CIE diagram at different Excitation wavelengths. Luminescence decay curves for Pr^3+^ emission at f) λ_ex_ = 275 nm and λ_em_ = 487 nm and g) λ_ex_ = 275 nm and λ_em_ = 620 nm. Luminescence decay curves for Er^3+^ emission at h) λ_ex_ = 380 nm and λ_em_ = 555 nm.

The PLE and emission spectra of CGGO:Pr, CGGO:Pr/Er and CGGO:Pr/Er/Yb phosphors under 275 nm UV light irradiation are presented in **Figure** **5**a,b, respectively. The characteristic excitation and emission bands due to different transitions of Pr^3+^ ions were observed in the codoped CGGO phosphors. The inset in Figure 5b displays the Pr^3+^ emission images of the respective phosphors. The PLE spectra recorded at 555 nm shows the excitation bands due to both the Er^3+^ and Pr^3+^ ions (Figure 5c). The 4f ⟶ 4f transitions of Er^3+^ ions were observed in the 350–450 nm regions with intense absorption at 380 nm due to ^4^I_15/2_ → ^2^G_11/2_ transition.^[^ ^56^ ^]^ Under 380 nm excitation, the emission spectra (Figure 5d) display the characteristic green and red emission bands of Er^3+^ ions in CGGO:Pr/Er and CGGO:Pr/Er/Yb phosphors (inset shows emission image). The sharp peaks at 524, 555, and 678 nm can be assigned to the ^2^H_11/2_ → ^4^I_15/2_ and ^4^S_3/2_ → ^4^I_15/2_, ^4^F_9/2_ → ^4^I_15/2_ transition of Er^3+^, respectively.^[^ ^57^ ^]^ After Yb^3+^ codoping, the luminescence intensity of Er^3+^ was significantly enhanced in CGGO:Pr/Er/Yb phosphor. The CIE color coordinate diagram in Figure 5e displays the multicolor emission in codoped CGGO phosphors under 275 and 380 nm UV light irradiation. The color coordinates of Pr^3+^ emission are (0.408, 0.392), (0.415, 0.394), and (0.422, 0.389) in CGGO:Pr, CGGO:Pr/Er and CGGO:Pr/Er/Yb phosphors under 275 nm excitation, respectively. The color coordinates of Er^3+^ green emission are (0.272, 0.574) and (0.281, 0.596) in CGGO:Pr/Er and CGGO:Pr/Er/Yb phosphors under 380 nm excitation, respectively.

The luminescence decay curves of Pr^3+^ and Er^3+^ are presented in Figure 5f–h recorded at different excitation and emission wavelengths. The luminescence decay curves of Pr^3+^ (λ_ex_ = 275 nm and λ_em_ = 487/620 nm) were best fitted into a three‐order exponential function (Equation (3) and decay curves of Er^3+^ (λ_ex_ = 380 nm and λ_em_ = 555 nm) were fitted by a bi‐exponential function as follows:

(4) $$I\left(t\right)=A_{1}\exp\left(-t/\tau_{1}\right)+A_{2}\exp\left(-t/\tau_{2}\right)$$

where, “I(t)” represents the Er^3+^ luminescence intensity, τ_1 _and τ_2_ are fitted lifetime components, and A_1_ and A_2_ denote pre‐exponential constants. The bi‐exponential decay of Er^3+^ emission indicated the existence of additional de‐excitation pathways such as multiphonon relaxation and cross‐relaxation in addition to the radiative transition. The lifetime values decreased for both the ^1^D_2_ state (620 nm) and ^3^P_0_ state (487 nm) of Pr^3+^ ions on codoping of Er^3+^ and Er^3+^/Yb^3+^ which induced non‐radiative energy transfer (ET) processes between dopant ions and resulting in faster decay. The Er^3+^ lifetime do not vary much in CGGO:Pr/Er and CGGO:Pr/Er/Yb phosphors.

**Figure 6:**
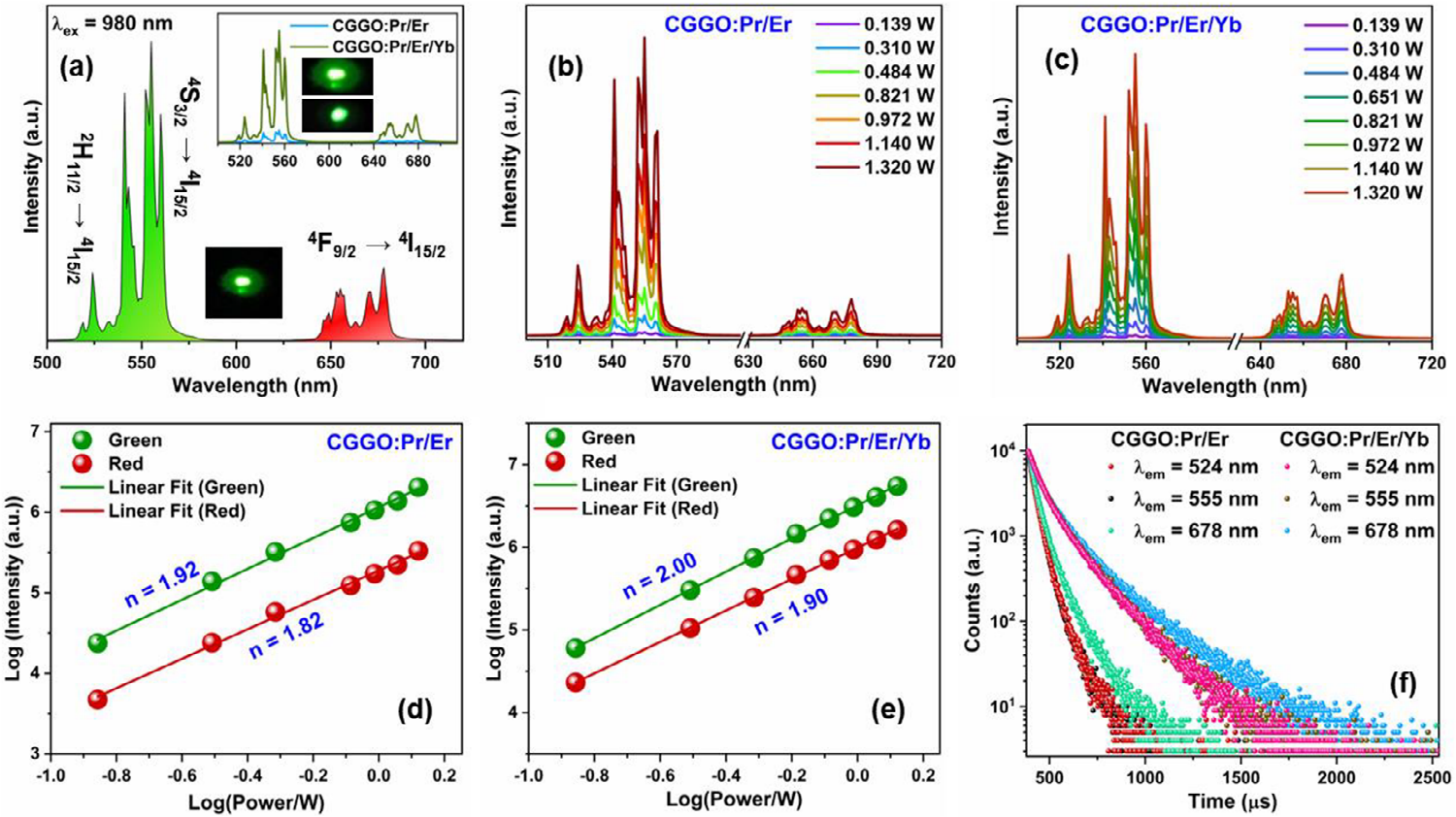
a) UCL spectra of Er^3+^ ions in CGGO:Pr/Er/Yb phosphor (inset shows UC emission spectra with and without Yb^3+^ codoping). b–e) Laser power‐dependent UC luminescence and logarithmic plots of UCL and laser power of CGGO:Pr/Er and CGGO:Pr/Er/Yb phosphor, respectively. f) Decay curves were monitored at 524, 555, and 678 nm in Er^3+^ codoped phosphors.

**Figure** **6**a shows the UCL spectra of Er^3+^‐codoped phosphors under 980 nm laser irradiation at a laser power of 0.821 W. The sharp emission peaks at ≈524 and 555 nm correspond to the ^2^H_11/2_ → ^4^I_1__5/2_ and ^4^S_3/2_ → ^4^I_1__5/2_ transitions of Er^3+^ ions and the sub‐bands are observed due to stark splitting of ^2^H_11/2_/^4^S_3/2_ energy levels.^[^ ^15^, ^19^ ^]^ The weak red emission peaked at 678 nm is due to the ^4^F_9/2_ → ^4^I_15/2_ transition of Er^3+^. On Yb^3+^ codoping in CGGO:Pr/Er phosphor, both the green and red UCL intensities increased significantly (inset of Figure 6a) which is also depicted by UCL emission images. This can be explained by improved absorption of 980 nm photons in CGGO:Pr/Er/Yb phosphor and ET from Yb^3+^ to Er^3+^ ions. The color coordinates of Er^3+^ UCL emission are (0.315, 0.672) and (0.321, 0.677) for CGGO:Pr/Er and CGGO:Pr/Er/Yb phosphors, respectively.

The laser power dependent UCL spectra for CGGO:Pr/Er and CGGO:Pr/Er/Yb phosphors show increased UCL intensity with increasing laser power from 0.139 to 1.32 W (Figure 6b,c). The non‐linear dependence of UCL on the laser power (P) can be expressed as follows:

(5) $$I\propto P^{n}$$

where n states the count of photons actively participating in UCL mechanisms and “I” denotes UCL intensity. The slope value (n) is evaluated by linear fitting of the experimental data in log(I) versus log(P) plots (in Figure 6d,e). The n values for CGGO:Pr/Er were calculated as 1.92 for green [^2^H_11/2_/^4^S_3/2_ → ^4^I_15/2_] and 1.82 for red (^4^F_9/2_ → ^4^I_15/2_) UCL which indicated that two photons are involved in UC mechanisms.^[^ ^66^ ^]^ The n values for CGGO:Pr/Er/Yb were increased to 2.00 for green and 1.90 for red UCL which can be explained by enhanced upconversion rate on Yb^3+^ codoping. The schematic energy level diagram showing Er^3+^ UC transitions and ET processes is presented in Figure S6 (Supporting Information). Under 980 nm laser radiation, the electrons in ^2^F_7/2_ energy level of Yb^3+^ ions get excited to the higher ^2^F_5/2_ state. Following this, the excited Yb^3+^ ions can transfer the energy to Er^3+^ ions by ET pathways and relax non‐radiatively to the ground state. Er^3+^ ions are pumped from the ^4^I_15/2_ ground state to the ^4^I_11/2_ excited level via ET process or ground state absorption (GSA). The simultaneous absorption of another photon by excited Er^3+^ ions populate the higher ^4^F_7/2_ energy level through another ET route or excited state absorption (ESA). The non‐radiative relaxation of Er^3+^ from the ^4^F_7/2_ level result in population of lower ^2^H_11/2_/^4^S_3/2_ and ^4^F_9/2_ energy levels. In addition, another ET pathway from Yb^3+^ to Er^3+^ can pump electrons to ^4^F_9/2_ state from ^4^I_13/2_ excited state. The excited Er^3+^ ions in ^2^H_11/2_/^4^S_3/2_ and ^4^F_9/2_ levels emit green (524/555 nm) and red (678 nm) photons on radiative relaxation to ^4^I_15/2_ level, respectively.

The luminescence decay curves of Er^3+^ UCL (λ_ex_ = 980 nm and λ_em_ = 524/555/678 nm) presented in Figure 6f were fitted by a bi‐exponential function (Equation (4)). The lifetime of ^2^H_11/2_ state (524 nm) increased from 46.5 to 127.3 µs, the lifetime of ^4^S_3/2_ state (555 nm) increased from 45.8 to 130.1 µs, and lifetime of ^4^F_9/2_ state (678 nm) increased from 63.9 to 180.9 µs on Yb^3+^ codoping in CGGO:Pr/Er phosphor. The increase in lifetime values of ^2^H_11/2_ /^4^S_3/2_ and ^4^F_9/2_ excited states increased on codoping of Yb^3+^ due to ET from Yb^3+^ to Er^3+^ ions.

### 2.5 PersL and Trap Engineering in Codoped CGGO:Pr Phosphors

**Figure 7:**
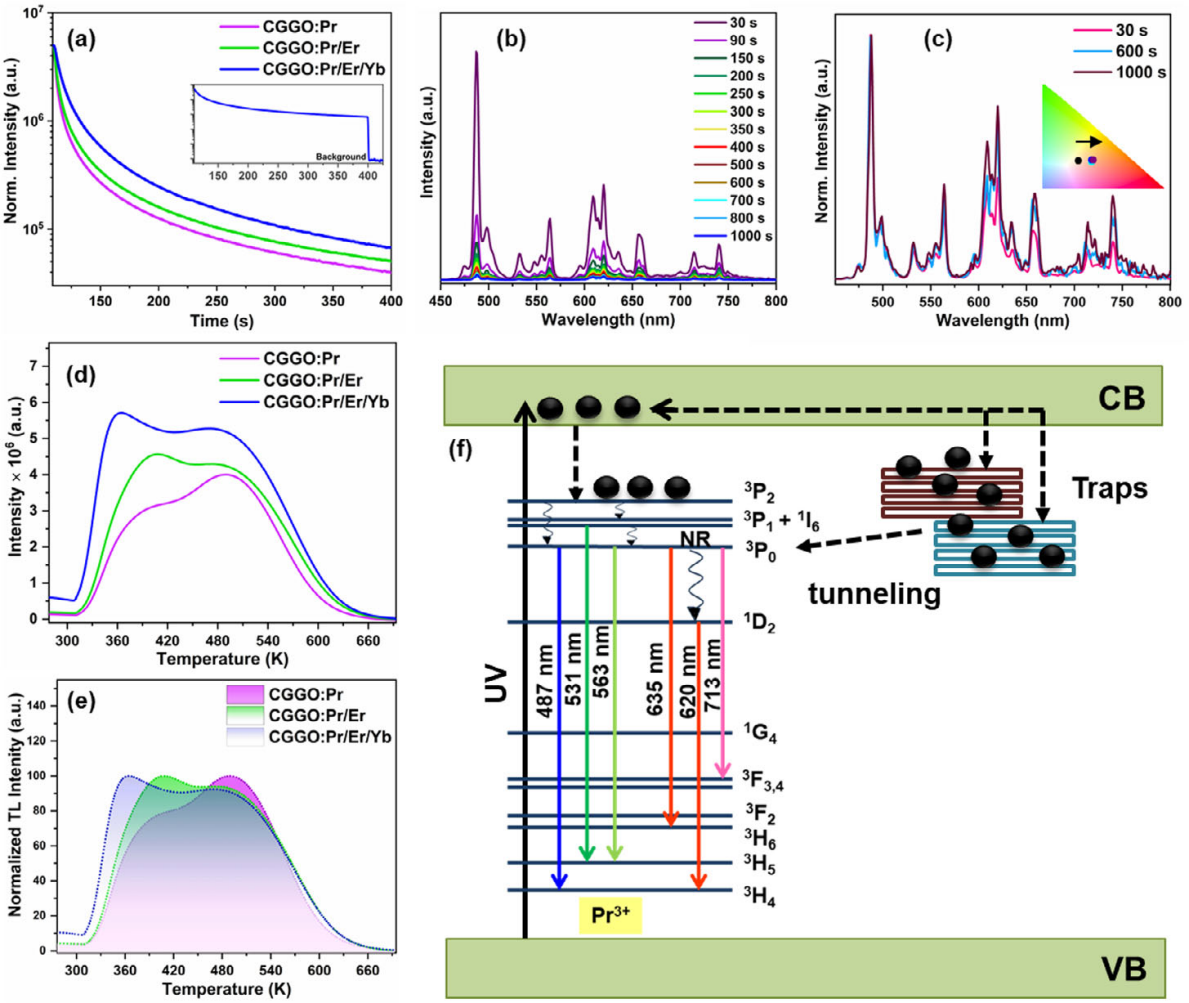
a) PersL decay curves of codoped CGGO phosphors monitored at 487 nm. b) Afterglow emission spectra of Pr^3+^ in CGGO:Pr/Er/Yb phosphor recorded at different intervals for 1000 s under 275 nm charging for 100 s. c) Partial normalized afterglow emission spectra. d) TL glow curve of codoped CGGO phosphors. e) Normalized TL glow curve. f) Schematic diagram illustrating the PersL mechanism of Pr^3+^ emission.

**Figure** **7**a presents the PersL decay curves of Pr^3+^ monitored at 487 nm in CGGO:Pr, CGGO:Pr/Er and CGGO:Pr/Er/Yb phosphors after charging with 275 nm for 100 s. The strong PersL of Pr^3+^ was observed and PersL increased significantly on Er^3+^ and Er^3+^/Yb^3+^ codoping in CGGO:Pr phosphor. Figure 7b shows the afterglow emission spectra of Pr^3+^ in CGGO:Pr/Er/Yb phosphors recorded at different intervals up to 1000 s after UV (275 nm) light irradiation for 100 s. The intensity of different transitions from the ^1^D_2_ and ^3^P_0_ state of Pr^3+^ exhibit distinct behavior with time (Figure 7c). The red emission intensity from ^1^D_2_ state increased relatively with time after ceasing the UV excitation which resulted in gradual shift of CIE color coordinates toward red spectral region (inset of Figure 7c).

To gain better insights about the nature and types of traps formed after doping in CGGO, the TL glow curves were recorded after charging of all samples with 275 nm UV light in the temperature range of 273–673 K with a heating rate of 2 K s^−1^ (Figure 7d). The broad TL curves were observed for all the codoped CGGO phosphors which confirmed the formation of various shallow and deep intermediate trap levels in‐between VB and conduction band (CB). Interestingly, the TL glow curves exhibit a clear shift in the peak maxima toward lower temperatures which indicated that Er^3+^ and Er^3+^/Yb^3+^ codoping favored the formation of shallower traps (Figure 7e). Moreover, the overall trap density increased significantly on codoping of Ln^3+^ ions as more defects are formed due to aliovalent doping. The trap depths (E) in all phosphors can be calculated by using the following relation:

(6) $$E=T_{m}/500$$

Here, T_m_ denotes the peak temperature (in K). To determine the different trap depths in doped CGGO phosphors, we performed Gaussian fitting of the TL spectra to obtain different T_m_ values (Figure S7a–c, Supporting Information). The TL glow curve of CGGO:Pr phosphor was deconvoluted into three emission peaks at 361 K (trap I), 395 K (trap II), and 495 K (trap III) with trap depths of ∼0.72, 0.79, and 0.99 eV, respectively. The Gaussian fitting of TL glow curve of CGGO:Pr/Er phosphor indicated similar trap depths as CGGO:Pr while the intensity of low temperature TL peaks increased significantly. This suggested that shallower traps are favorably formed on Er^3+^ codoping. In CGGO:Pr/Er/Yb phosphor, four TL emission peaks were obtained at 347 K (trap I), 376 K (trap II), 429 K (trap III), and 515 K (trap IV) with trap depths of ≈0.69, 0.75, 0.86, and 1.03 eV, respectively. This suggested that Yb^3+^ codoping induced increased formation of shallower traps along with the formation of deeper traps. The experimentally calculated trap depths correspond well with the defect states evaluated by DFT calculations for oxygen and cation vacancies. This clearly indicated that Er^3+^ and Yb^3+^ codoping induced the formation of additional electron acceptor trap states below CB with shallow trap depths. The optimum trap depth desired for strong PersL at room temperature must lie between 0.65 and 0.75 eV. The codoping of Er^3+^ and Yb^3+^ ions in CGGO:Pr resulted in effective trap engineering and enhanced the formation of defect levels with optimum trap depths (≈0.69 to 0.75 eV) which resulted in enhanced PersL at room temperature.

The TL glow curve of CGGO:Pr phosphor after X‐ray charging was deconvoluted into two emission peaks at 438 K (trap I) and 501 (trap II) with trap depths of ≈0.87 and 1.00 eV, respectively (Figure S8a, Supporting Information). With X‐ray charging, higher concentration of deeper traps is filled with electrons. The PersL decay curve of CGGO:Pr phosphor after ceasing X‐ray excitation exhibit the Pr^3+^ afterglow for ≈1 h (Figure S8b, Supporting Information). The PersL mechanism on X‐ray charging involves similar trapping and releasing processes of electrons shown in Figure 7f.

**Figure 8:**
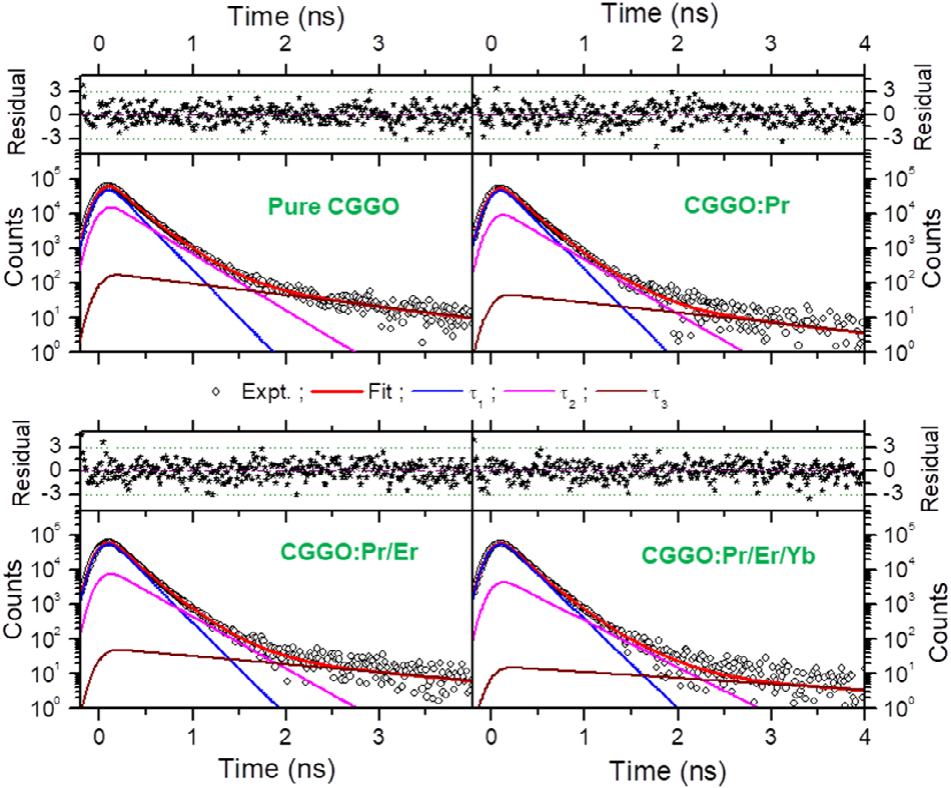
PALS spectra show evaluation of the individual lifetimes using PALSfit.

The advanced PALS technique was employed to further probe the density and types of intrinsic defects and the defects formed on Pr^3+^, Er^3+^, and Yb^3+^ codoping in CGGO matrix. Positron annihilation lifetime spectra in all the samples could be fitted to sum of three lifetime components using PALSfit3 as shown in **Figure** **8**. Various components of the lifetime spectra as well as quality of fits are also shown in the Figure 8. The summary of the positron lifetimes and the corresponding intensities obtained are given in Table S2 (Supporting Information). The salient observations from the table are that all the positron lifetimes increased with doping while the intensity of first positron lifetime increased at the expense of the other. The longest lifetime (τ_3_) in the range of ns is well known in the powder samples due to positronium formation on the surface of the particles when positron lifetimes are measured on powder samples. Systematic reduction in the intensity with increase in lifetime in this case may be due to better grain grown in the doped samples. Among the other two positron annihilation lifetimes, the shorter lifetime (τ_1_) is due to positron annihilations in the bulk along with positron annihilation in shallow traps whose lifetime is indistinguishable from bulk lifetime. The second lifetime (τ_2_) is due larger vacancy clusters near the surface regions and with positron annihilations on the surface on the grains. The reduction in the intensity of second component along with increase in the lifetime suggests that smaller fraction of positrons are reaching the surface/trapped in larger vacancy clusters. Gradual increase in first positron lifetime and intensity suggest that positrons are experiencing many shallow positron traps. For positrons, monotonic increase in intensity also suggests that these vacancies could be having trapped electrons.

The PALS results are in well correlation with the trap tuning observed in TL study. Based on the DFT, PALS, and TL results, it can be proposed that oxygen vacancies are formed as intrinsic defects in CGGO matrix due to the lowest defect formation energy. On doping of aliovalent Pr^3+^, the V_Ca_ and Pr_Ca_^•^ defects will form due to charge mismatch. The DFT studies indicated the location of these defects near VB with very high defect formation energy (Table 1) and hence, V_Ca_′′ formation is not much feasible. The Pr_Ca_^•^ defect levels can act as electron trap centers during UV excitation and lie near CB, and V_Ca_′′ will act as hole trap centers.^[^ ^21^, ^29^ ^]^ The codoping of Er^3+^ and Yb^3+^ ions will result in enhanced formation of electron acceptor traps, Yb_Ca_^•^ and Er_Ca_^•^ in higher concentration. As indicated by PALS studies, the vacancies formed on codoping are having trapped electrons with shallower trap depths that may be the Yb_Ca_^•^ and Er_Ca_^•^ defects levels. Figure 3i shows that different charged oxygen vacancies lie at different trap depth near CB. Hence, the TL glow curve in CGGO:Pr is mainly composed of V_O_ trap levels with wide trap depths. On codoping, the shallower Yb_Ca_^•^ and Er_Ca_^•^ trap levels are formed in relatively higher concentrations and resulted in trap tuning to optimum trap depths for strong PersL at room temperature.

The schematic illustration of PersL mechanism and energy level diagram of Pr^3+^ ions showing the different 4f ⟶ 4f transitions and trap assisted PersL is presented in Figure 7f. The shallow and deep traps can capture electrons during the UV excitation at 275 nm. After ceasing UV light irradiation, the electrons in the traps can be released slowly by thermal agitation and result in population of Pr^3+^ excited states that induce long afterglow emissions. The electrons from deep trap levels are released at a slower rate by quantum tunneling.

### 2.6 Applications

#### 2.6.1 X‐Ray Imaging

To demonstrate the potential of organic‐inorganic hybrid thin films based on CGGO:Pr phosphor for high‐quality X‐ray imaging applications, the PMMA–CGGO:Pr composite film was fabricated with 50 wt.% loading of phosphor into PMMA matrix.

**Figure 9:**
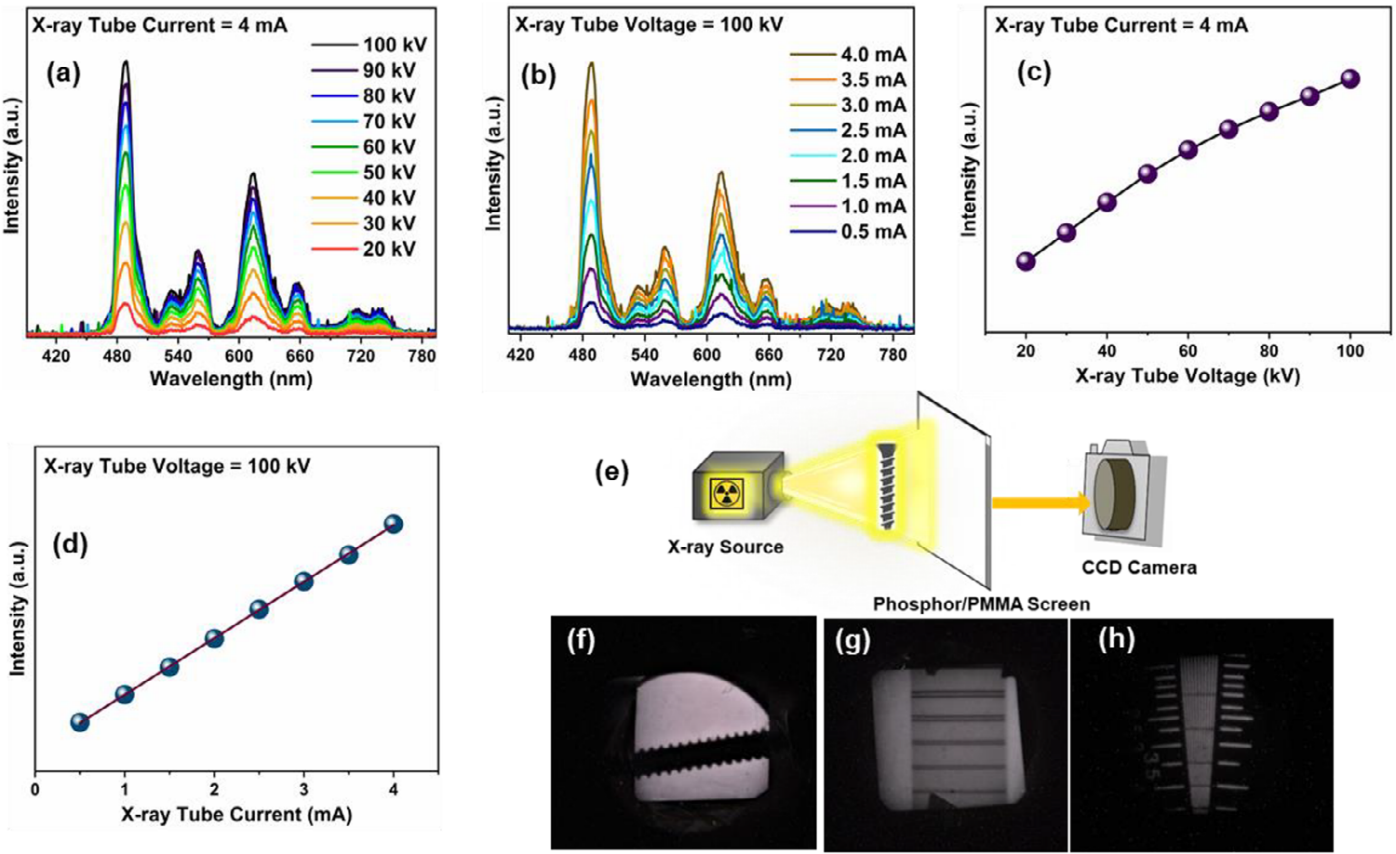
RL spectra of PMMA‐CGGO:Pr film as a function of a) X‐ray tube voltage and b) X‐ray tube current. c,d) Integrated RL intensity trend of PMMA‐CGGO:Pr film with varying X‐ray tube voltage and current, respectively. e) X‐ray imaging set‐up. f) X‐ray imaging of a screw on composite screen. g) Radiographic image formed by a duplex wire (4 lp mm^−1^). h) Images formed on composite screen using a SS line mask.

The radioluminescence (RL) characteristics of PMMA–CGGO:Pr composite film was measured at different X‐ray tube current and voltage as shown in **Figure** **9**a,b. The intense RL peak from ^3^P_0_ level was observed at 487 nm which is in line with the emission spectra under UV excitation. The variation in RL intensity of the PMMA–CGGO:Pr composite film as a function of X‐ray tube voltage (kV) and current (mA) is shown in Figure 9c,d, respectively. The RL intensity initially increases exhibits a linear dependence and get saturated at higher voltage values >70 kV. Moreover, the RL intensity shows a linear dependence on as a function of X‐ray tube current from 0.5 to 4 mA. The linear dependence of RL output of the PMMA–CGGO:Pr composite film supported the potential of this hybrid composite film for applications as X‐ray screens and radiation detection.^[^ ^67^ ^]^

Furthermore, the X‐ray imaging potential of organic‐inorganic hybrid PMMA‐CGGO:Pr screens was evaluated using the X‐ray imaging setup shown in Figure 9e. The composite film was used for the imaging of a screw placed in front of X‐ray source on the composite scintillator screen in the transmission mode and X‐ray images were captured by a CCD camera kept behind the screen. Figure 9f–h presents the X‐ray images produced using the hybrid PMMA‐CGGO:Pr screen. A spatial resolution of 130 microns and a resolution of 4 lp mm^−1^ was achieved with the PMMA‐CGGO:Pr film as shown in the radiography image of a duplex wire. The line profiles of flat‐field corrected X‐ray image of lead lined resolution pattern (Figure S9, Supporting Information) at tube settings of 100 kV and 4 mA revealed the contrast of 5.3% at 3.75 lp mm^−1^ and 4.8% at 4.2 lp mm^−1^, respectively. This indicated the high resolution of X‐ray imaging using the PMMA‐CGGO:Pr composite film. The X‐ray images under different X‐ray tube current and voltage settings are provided in Figure S10 (Supporting Information) to demonstrate that the X‐ray imaging can be performed at a lower X‐ray energy using the PMMA‐CGGO:Pr composite film. The RL intensity of PMMA‐CGGO:Pr composite film was monitored for continuous irradiation of ≈15 min at 100 kV and 4 mA X‐ray tube power setting (Figure S11, Supporting Information). It shows that the film is radiation stable for X‐ray imaging (non‐destructive technique) applications. The resolution of composite film is higher than the resolution of 2.8 lp mm^−1^ reported earlier for commercially used Gd_2_O_2_S:Tb and GOS:Tb X‐ray screens.^[^ ^68^ ^]^

#### 2.6.2 Multimodal Anticounterfeiting

**Figure 10:**
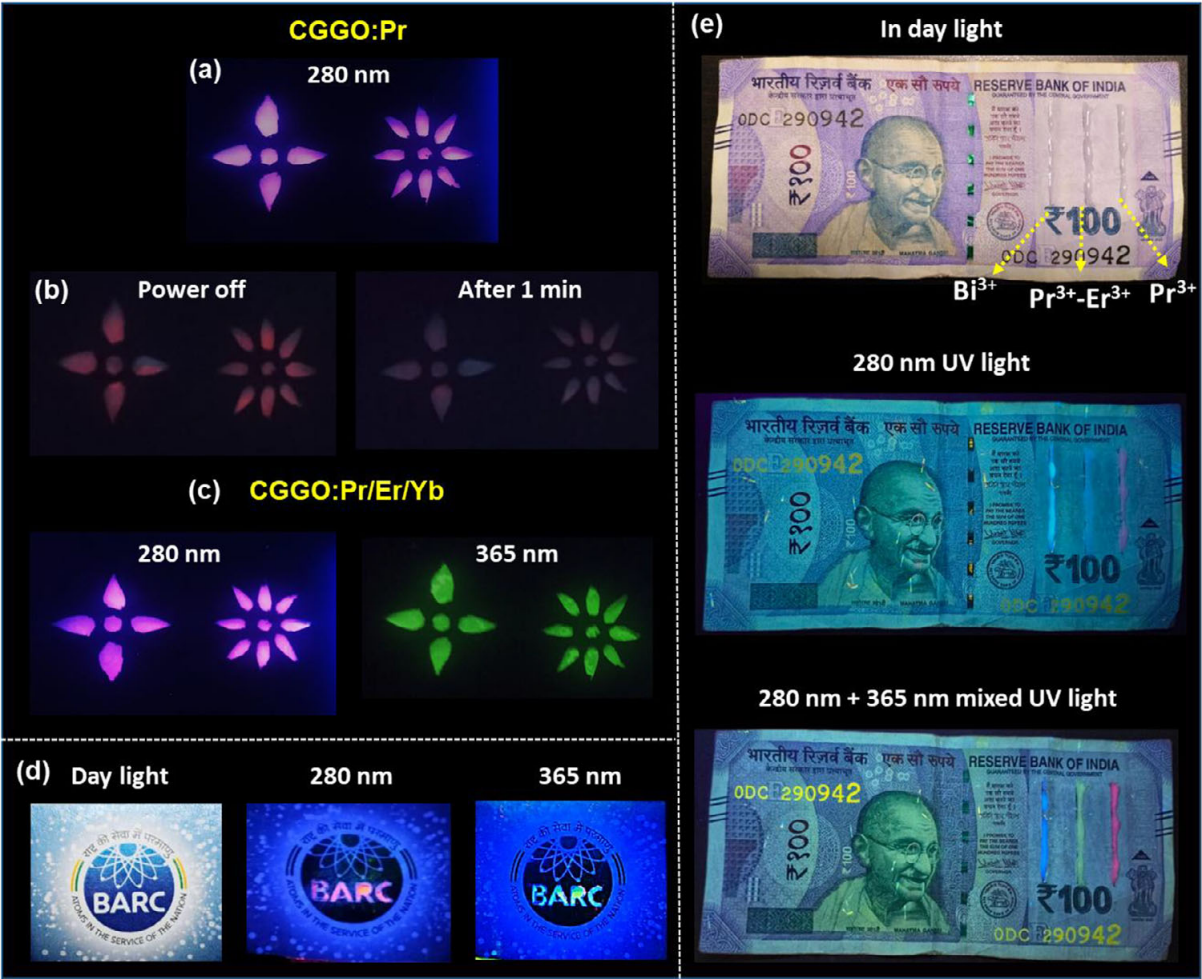
Digital photographs of different anti‐counterfeiting labels with a) CGGO:Pr phosphor under 280 nm UV light and b) after irradiation stops. c) Digital photographs of different anti‐counterfeiting labels with codoped CGGO:Pr/Er/Yb phosphor under 254  and 365 nm UV lights. d) Logo of the institution made with Pr^3+^‐Er^3+^ codoped phosphor in daylight, under 280 nm, and under 365 nm UV light. e) Phosphor line coated Indian rupee note in day light under 280 nm UV light, and under 280 nm + 365 nm mixed UV light.

To examine the real‐world security usage of the obtained blue, red, and green phosphors as anti‐counterfeiting patterns, security inks were prepared by dispersing the powder in the polyacrylic polyols for obtaining transparent viscous solutions. Different structures, an institute logo, and a currency note are painted with the prepared ink. The CGGO:Pr based patterns shows pinkish‐white emission under 280 nm LED light (**Figure** **10**a) Interestingly, when the UV light is switched off, the CGGO:Pr phosphor coated patterns still show the characteristic emission color which is sustained until one min due to the PersL property of the Pr^3+^‐doped system, as shown in Figure 10b. Similar CGGO:Pr/Er/Yb based patterns shows the pinkish‐white and green emission under 280 and 365 nm UV lights, respectively (Figure 10c). This unique feature of the codoped phosphors can be used to protect documents from acts of scam and falsification and also to avoid replication of logos and currency notes, etc. As seen in Figure 10d, the color of “BARC” is almost hidden in the daylight, however, it is glowing in red and greenish under 280 and 365 nm UV light. Similarly, these two phosphors were linearly coated on an Indian currency note along with a CGGO:Bi^3+^ phosphor with characteristic blue emission. Under daylight, the linear coating is almost invisible, however, when the 280 nm UV light and 280 nm + 365 nm mixed UV light was applied, the linear emissive marking are clearly visible (Figure 10e). These patterning concepts demonstrate that anti‐counterfeiting labels based on blue, red, and green phosphors offer exceptional security features. They remain inconspicuous under normal light conditions while displaying clear luminescent colors under UV light.

## 3 Conclusion

In this work, a single CGGO:Pr phosphor‐based system was designed to achieve multimodal emissions through a codoping approach involving Pr^3+^ and Er^3+^ ions in the CGGO garnet structure. Under various excitation wavelengths (275 and 380 nm), the Pr^3+^ and Er^3+^ ions exhibited strong whitish and green emissions with distinct peaks detected in both the visible and NIR spectral ranges. EXAFS analysis indicated that Pr^3+^ ions occupy two different sites: Ca^2+^ and Ga^3+^, which was further validated by hybrid DFT calculations. A prolonged PersL of Pr^3+^ ions was demonstrated after being charged with UV light (275 nm) for 100 s, and the codoping of Er^3+^/Yb^3+^ ions led to a significant enhancement in the PersL intensity. The TL and PALS studies revealed a significant increase in trap density, while trap depths were optimized from 0.984 eV to an ideal range of ≈0.73 eV through an aliovalent codoping method using Er^3+^/Yb^3+^. This optimization can be explained by the formation of new Yb_Ca_^•^ and Er_Ca_^•^ electron trap levels in addition to V_O_ levels. Further, the composite PMMA‐CGGO:Pr phosphor films demonstrated X‐ray imaging potential with a better resolution of 4 lp mm^−1^ than commercial Gd_2_O_2_S:Tb screens (2.8 lp mm^−1^). Additionally, the smart tridoped‐Pr^3+^/Er^3+^/Yb^3+^:CGGO phosphor exhibits three‐dimensional visible emissions under distinct excitation wavelengths of 275  and 380 nm, along with UC emissions under 980 nm laser irradiation. Together, these properties highlight the potential of the Pr^3+^ and Er^3+^‐codoped CGGO system for robust and high‐level anti‐counterfeiting coding. The afterglow emissions of Pr^3+^ following the cessation of UV light excitation are particularly promising for designing complex anti‐counterfeiting coding patterns for documents, currency, pharmaceuticals, and other industrial products.

## 4 Experimental Section

### Synthesis Method

The Ca_3_Ga_2_Ge_3_O_12_:0.03Pr^3+^ (x = 1.0 mol %) sample was synthesized using the high‐temperature solid‐state reaction method. The stoichiometric amounts of precursors, CaCO_3_, Ga_2_O_3_, GeO_2_, and Pr_6_O_11_ were mixed evenly by grinding for 20 min in a mortar pestle. The powder samples were heated at a temperature of 900 °C for 4 h followed by 1200 °C for 12 h. After intermittent grinding, the powder samples were annealed at 1350 °C for 6 h to obtain pure phase CGGO:0.03Pr^3+^ (CGGO:Pr) material. For the synthesis of CGGO:0.03Pr^3+^/0.03Er^3+^ (CGGO:Pr/Er) and CGGO:0.03Pr^3+^/0.03Er^3+^/0.02Yb^3+^ (CGGO:Pr/Er/Yb), the similar synthesis method was followed along with the addition of stoichiometric amounts of Er_2_O_3_ and Yb_2_O_3_, respectively. The powder material was ground well for further characterization and the details are provided in Sections S1 and S2 (Supporting Information).

### Fabrication of Composite PMMA‐CGGO:Pr Composite Film

The PMMA polymer was dissolved in a toluene solution with continuous stirring and heating at 40 °C. The required amount of CGGO:Pr^3+^ sample was dispersed in PMMA solution with ultrasonication for 1 h. Then, the thin film of PMMA‐CGGO:Pr^3+^ was fabricated by drop casting the solution on a glass plate and drying overnight.

## Conflict of Interest

The authors declare no conflict of interest.

## Supporting information



Supporting Information

## Data Availability

The data that support the findings of this study are available from the corresponding author upon reasonable request.
