# Supplementary material for: Unleashing the Potential of Defect Engineered Persistent Pr3+‐Activated Phosphors for Multi‐Dimensional Anti‐Counterfeiting and X‐Ray Imaging Applications
Source: Small. 2025 Apr 24;21(46):2501752. doi: 10.1002/smll.202501752 (PMC12632419; doi:10.1002/smll.202501752)
Supplement: Supplementary file 1 — Supporting Information [file SMLL-21-2501752-s001.docx]

**Supplementary Information**

**Unleashing the Potential of Defect Engineered Persistent Pr^3+^-Activated Phosphors for Multi-Dimensional Anti-counterfeiting and X-ray Imaging Applications**

*Annu Balhara,^1,2^ Santosh K. Gupta,^1,2*^ Partha Sarathi Ghosh,^2,3^ Malini Abraham,^4,5^ Mohit Tyagi,^2,6^Ashok Kumar Yadav,^7^ Subrata Das,^4,5^ Kathi Sudarshan,^1,2^ and P. S. Sarkar,^6^*

*^1^Radiochemistry Division, Bhabha Atomic Research Centre, Trombay, Mumbai-400085, India*

*^2^Homi Bhabha National Institute, Anushakti nagar, Mumbai-400094*

*^3^Glass & Advanced materials Division, Bhabha Atomic Research Centre, Trombay, Mumbai-400085, India*

*^4^Materials Science and Technology Division, CSIR-National Institute for Interdisciplinary Science and Technology, Thiruvananthapuram, Kerala-695019*

*^5^Academy of Scientific and Innovative Research (AcSIR), Ghaziabad-201002, India*

*^6^Technical Physics Division, Bhabha Atomic Research Centre, Mumbai-400085, India*

*^7^Atomic & Molecular Physics Division, Bhabha Atomic Research Centre, Mumbai – 400085*

**To whom correspondence should be addressed. Electronic mail: santoshg@barc.gov.in*

***S1. Characterization***

The powder XRD patterns were acquired on a Proto-AXRD bench top system in the 2θ range of 15-80° and a scan rate of 1°/min. Fourier Transform Infrared Spectroscopy (FTIR) was performed with a diamond ATR mode on a Bruker Alpha FTIR spectrometer in the scanning range from 500 to 4000 cm^-1^. Field emission scanning electron microscopy (FE-SEM) and energy-dispersive spectroscopy (EDS) analysis were performed on a Carl Zeiss, Model: GEMINISEM300. X-ray photoelectron spectroscopy (XPS) was carried out on PHI 5000 Versa Prob II, FEI Inc. The PL and PLE spectra were recorded with a continuous Xenon lamp (450 W) source on a FLS-1000 fluorescence spectrometer (Edinburgh Instruments, U.K.), and visible-PMT as the detector. Emission photographs were captured under 365 nm UV lamp by a canon camera.

An X-ray Absorption Spectroscopy (XAS) measurement, which comprises of both X-ray Near Edge Structure (XANES) and Extended X-ray Absorption Fine Structure (EXAFS) techniques, have been carried out on doped CGGO at RE L3-edges in fluorescence mode. The XAS measurements have been carried out at the Energy-Scanning EXAFS beamline (BL-9) at the Indus-2 Synchrotron Source (2.5 GeV, 100 mA) at Raja Ramanna Centre for Advanced Technology (RRCAT), Indore, India.**^1, 2^**

Positron annihilation lifetime spectra were acquired on powder samples with Na-22 as positron source and a lifetime spectrometer constructed from two identical BaF2 detectors where the signals were digitized using DDRS4PALS.**^3^** The spectrometer has overall time resolution of 216 ps. The positron annihilation lifetime spectra were analysed using PALSfit3 software.**^4^** For recording the image after X-ray irradiation, a homemade black box having monochrome CCD was used.

***S2. Computational details***

To examine the effect of various vacancy defects on the electronic structure and PL properties of Ca_3_Ga_2_Ge_3_O_12_, plane-wave based spin polarized density functional theory (DFT) calculations are performed using Vienna ab initio simulation package (VASP).**^5, 6^** The interaction between ions and electrons are described using the projector augmented wave (PAW) potentials.**^7^** The exchange correlation energies are described using the generalised gradient approximation (GGA) as parameterized by Perdew-Burke-Ernzerhof (PBE).**^8^** A 600 eV is taken into consideration as the kinetic energy cut-off for the electronic self-consistent field iterations. Additionally, every structure underwent optimisation until the force tolerance is less than 0.02 eV/Å, and the difference value of the total energy is less than 10^-5^ eV. The Brillouin-zone integrations are performed using a 5×5×5 Monkhorst-Pack k-point mesh.**^9^**

All the structural optimizations are performed using GGA-PBE functional and the density of states calculations are performed using hybrid DFT of Heyd, Scuseria, and Ernzerhof (HSE)**^10^** to overcome the well-known short coming of electronic band-gap under prediction of GGA. In HSE calculations the values of mixing coefficient (α) and screening parameter (μ) are chosen to be 0.2 Å^−1^ and 0.25, respectively.


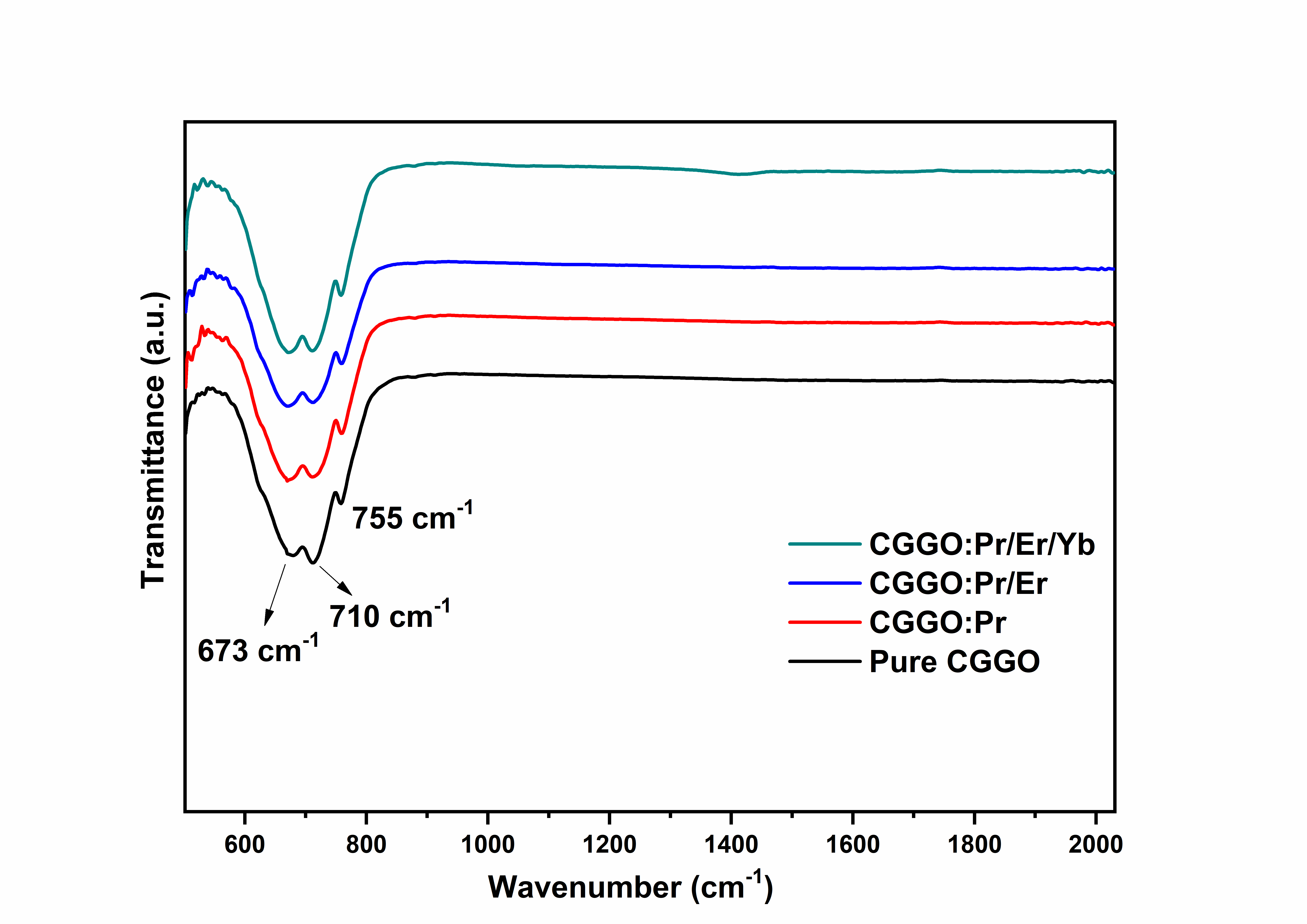


**Figure S1: FTIR spectra of pure CGGO and codoped CGGO samples.**


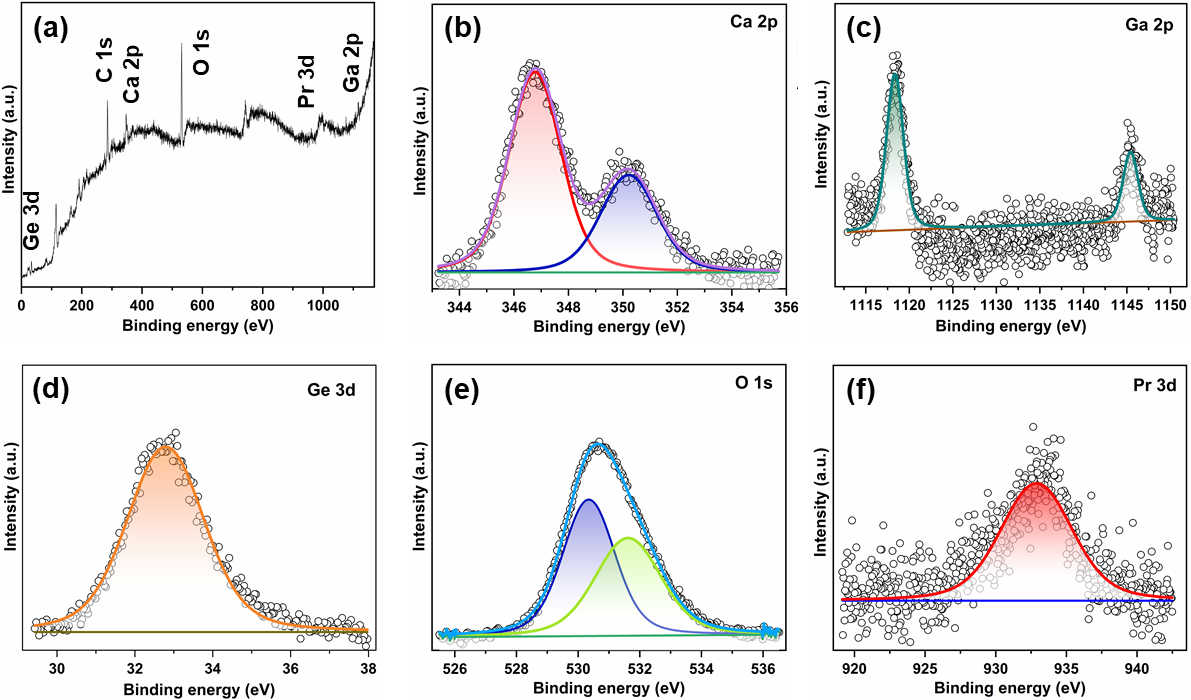


**Figure S2: (a) XPS survey spectra of CGGO:Pr sample. (b-f) XPS profiles of Ca 2p, Ga 2p, Ge 3d, O 1s, and Pr 3d, respectively.**

**Table S1: Bond lengths of different polyhedra in CGGO.**

| GaO_6_ | average Ga-O bond length=2.026 Å  polyhedral volume 11.04 Å^3^ |
| --- | --- |
| PrO_6_ | average Pr-O bond length=2.259 Å  polyhedral volume 15.30 Å^3^ |
| CaO_8_ | average Ca-O bond length=2.453 Å  polyhedral volume 25.19 Å^3^ |
| PrO_8_ | average Pr-O bond length=2.456 Å  polyhedral volume 25.38 Å^3^ |


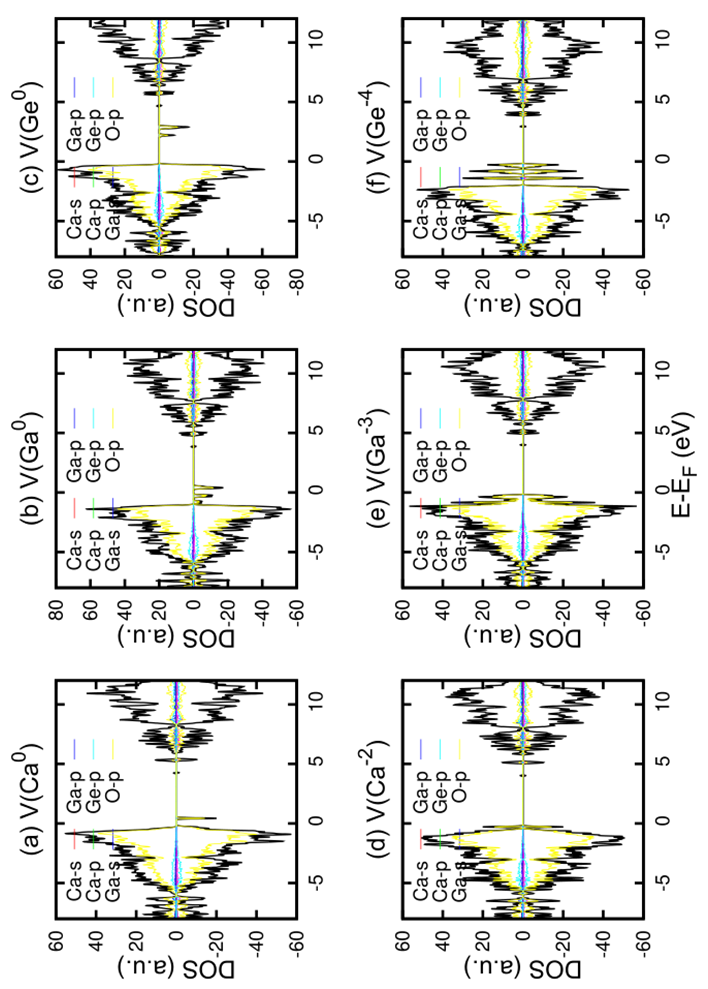


**Figure S3: DFT-HSE06 calculated total and angular momentum-decomposed DOS due to the presence of neutral Ge vacancy.**


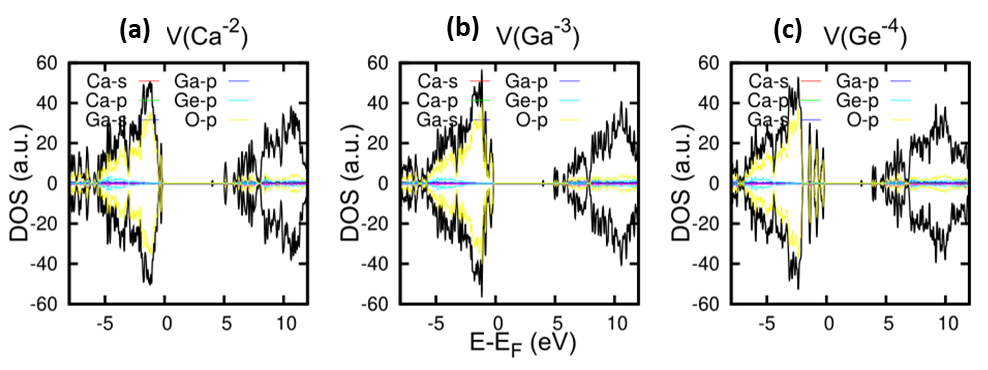


**Figure S4: DFT-HSE06 calculated total and angular momentum-decomposed DOS due to the presence of a (a) Ca vacancy with a charge of 2+ (VCa2+) (b) Ga vacancy with a charge of 3+ (VGa3+) and (c) Ge vacancy with a charge of 4+ (VGe4+).**

**
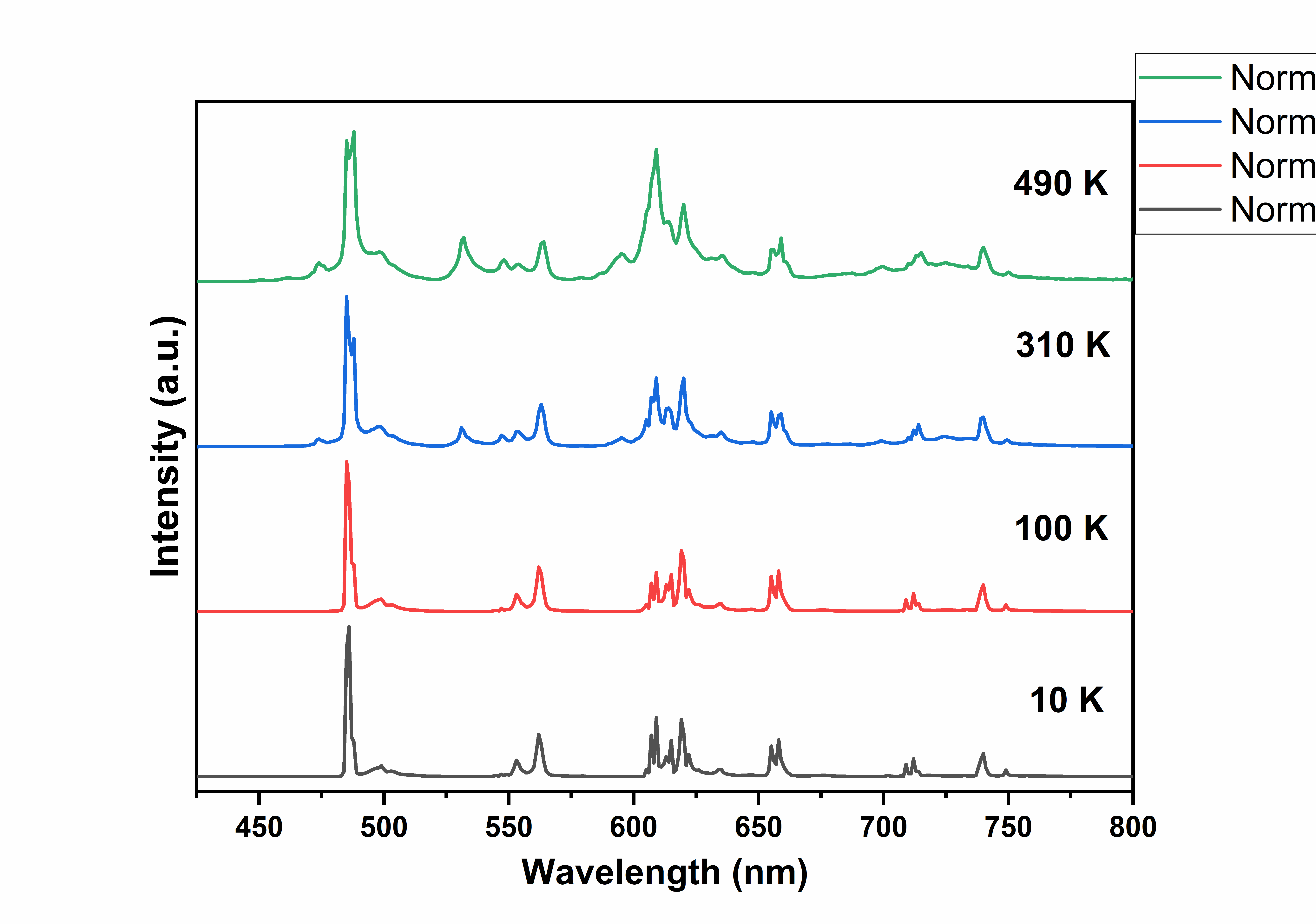
**

**Figure S5: Partial temperature-dependent emission spectra of CGGO:Pr sample under 275 nm excitation.**

**
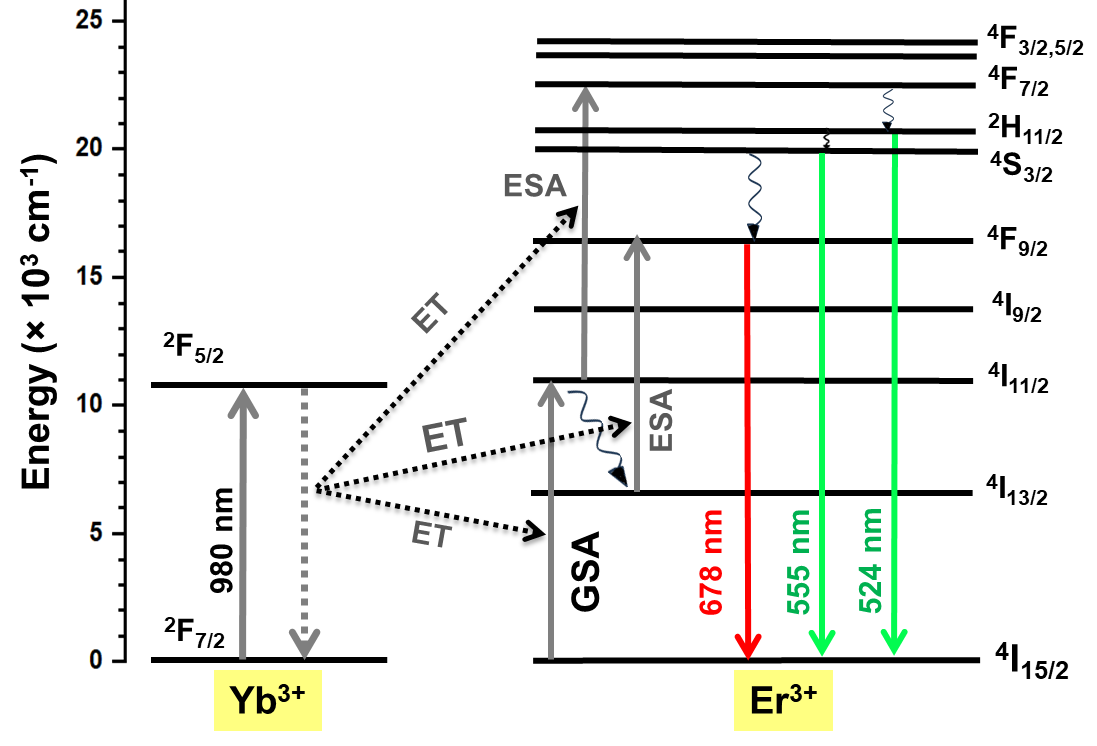
**

**Figure S6: Schematic energy level diagram showing Er^3+^ UC transitions and energy transfer.**

**
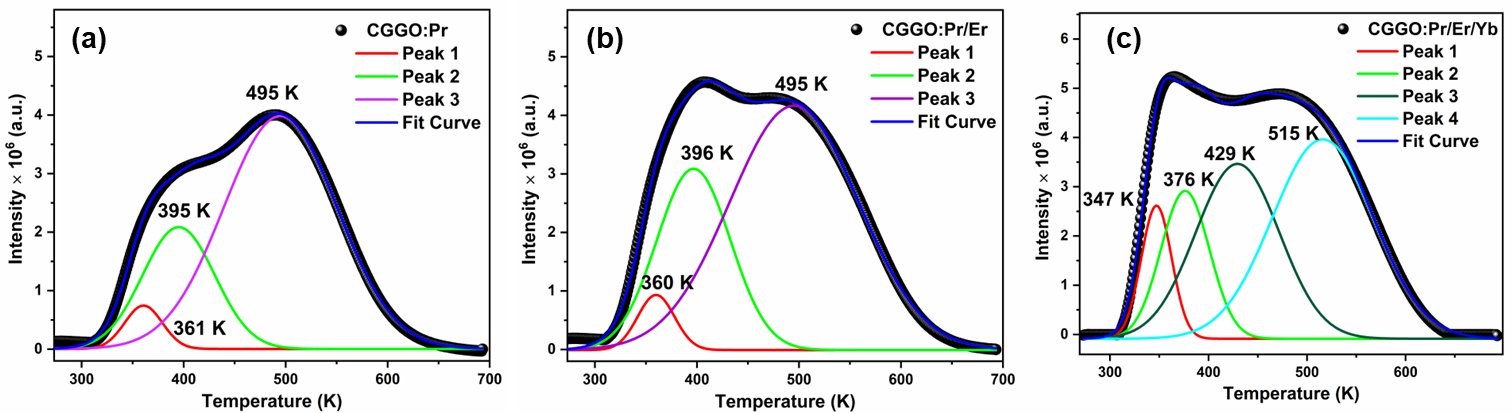
**

**Figure S7: The Gaussian fitting of the thermoluminescence spectrum of (a) CGGO:Pr, (b) CGGO:Pr/Er, and (c) CGGO:Pr/Er/Yb phosphor along with emission peak values.**

**
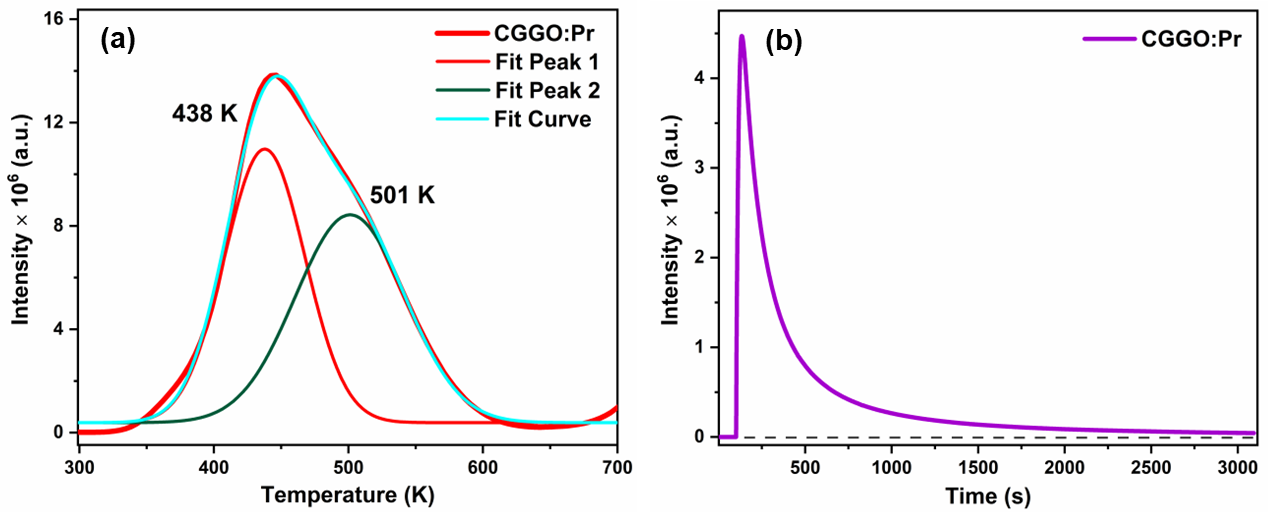
**

**Figure S8: (a) Gaussian fitting of the thermoluminescence spectrum of CGGO:Pr after X-ray irradiation for 100 s, (b) PersL decay curve of CGGO:Pr after X-ray excitation monitored at 487 nm.**

**Table S2: Summary of positron annihilation lifetimes and intensities in doped CGGO samples.**

| **Dopant** | **τ_1_ (ps)** | **I_1_ (%)** | **τ_2_ (ps)** | **I_2_ (%)** | **τ_3_ ( ns)** | **I_3_ (%)** |
| --- | --- | --- | --- | --- | --- | --- |
| Undoped CGGO | 159 ± 4 | 69 ± 5 | 267 ± 13 | 30 ± 5 | 1.33 ± 0.06 | 1.1 ± 0.1 |
| CGGO:Pr | 161 ± 3 | 78 ± 5 | 278 ± 16 | 21 ± 5 | 1.52 ± 0.18 | 0.38 ± 0.07 |
| CGGO:Pr/Er | 162 ± 2 | 83 ± 3 | 290 ± 14 | 16 ± 3 | 1.85± 0.10 | 0.44 ± 0.02 |
| CGGO:Pr/Er/Yb | 168 ± 2 | 89 ± 2 | 321 ± 19 | 10 ± 2 | 2.54 ± 0.33 | 0.19 ± 0.03 |

**
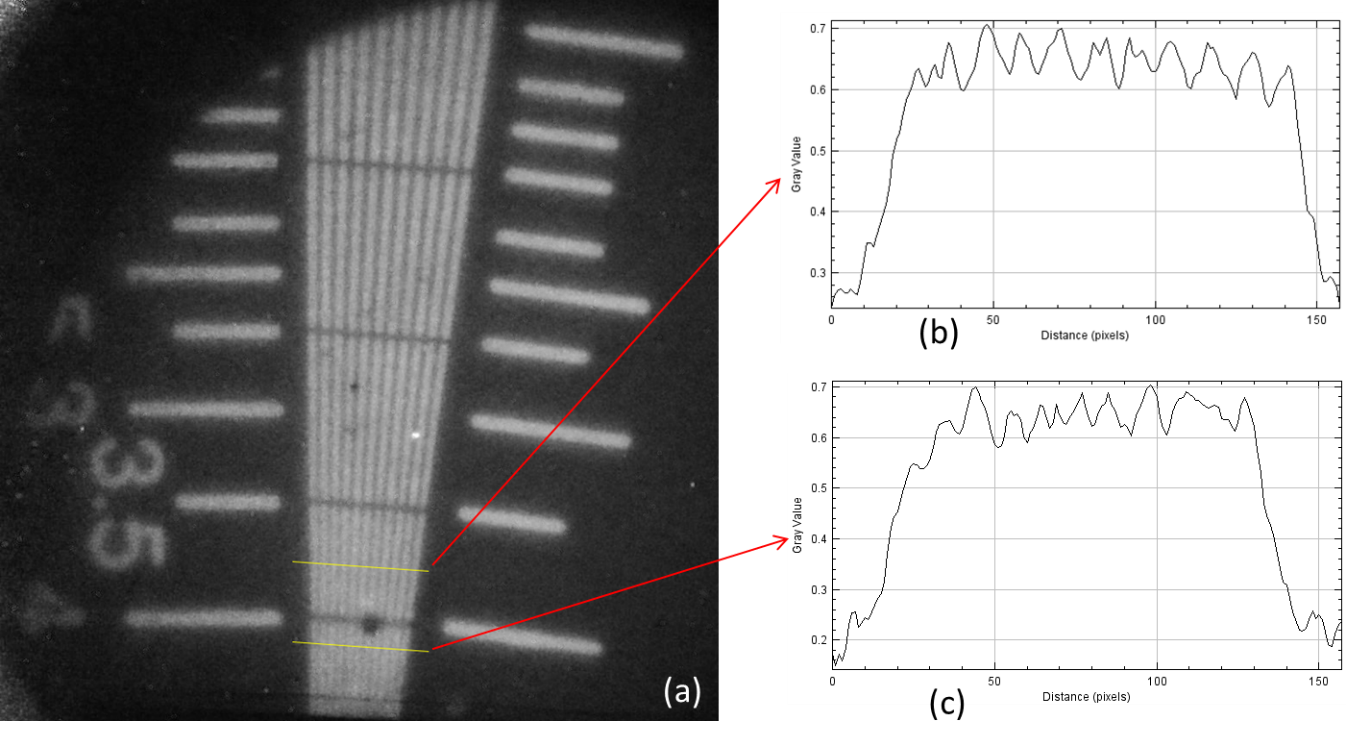
**

**Figure S9: Flat-field corrected X-ray image of lead lined resolution pattern at tube settings of 100 kV and 4 mA, (b) & (c) are the line profiles revealing the contrast of 5.3% at 3.75 lp/mm and 4.8% at 4.2 lp/mm respectively. Here, intensity gray values are normalized to 1.**

**Figure S10: X-ray images of lead lined resolution pattern at different power setting of X-ray tube.**

**
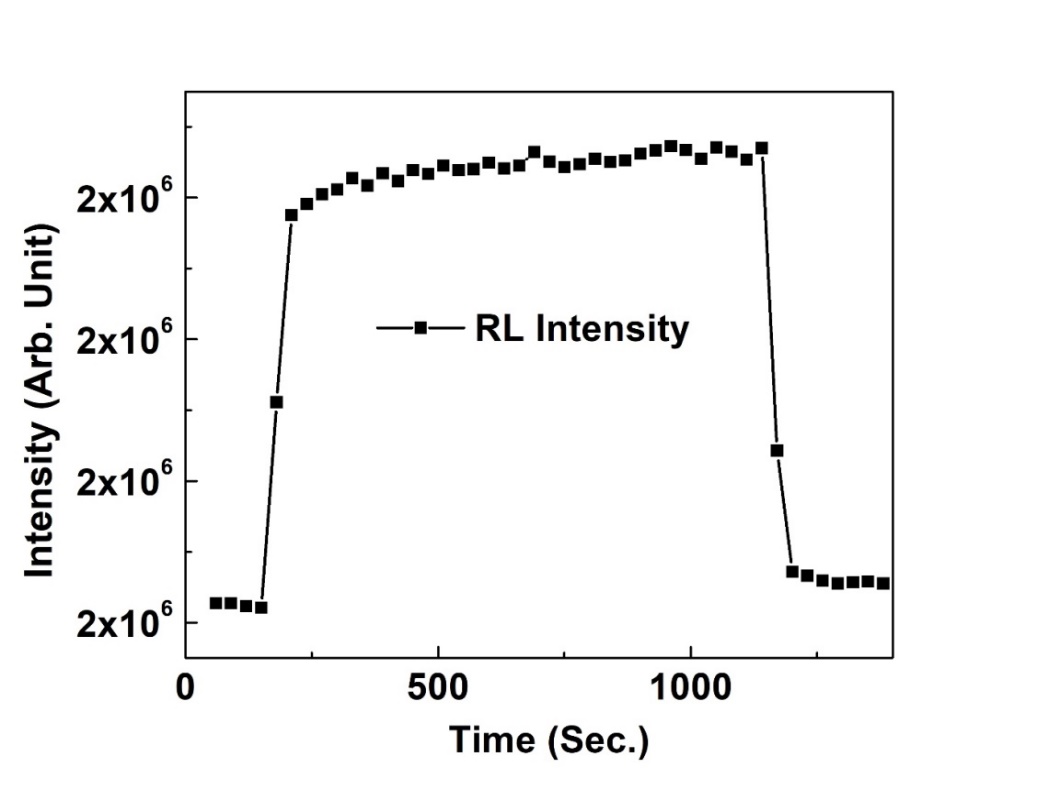
**

**Figure S11: Radio-luminescence intensity of polymer film composite for continuous irradiation of about 15 min at 100 kV and 4 mA tube power setting.**

**S3. References**

**1. Basu, S.; Nayak, C.; Yadav, A.; Agrawal, A.; Poswal, A.; Bhattacharyya, D.; Jha, S.; Sahoo, N. In *A comprehensive facility for EXAFS measurements at the INDUS-2 synchrotron source at RRCAT, Indore, India*, Journal of Physics: Conference Series, 2014; IOP Publishing: 2014; p 012032.**

**2. Poswal, A.; Agrawal, A.; Yadav, A.; Nayak, C.; Basu, S.; Kane, S.; Garg, C.; Bhattachryya, D.; Jha, S.; Sahoo, N. In *Commissioning and first results of scanning type EXAFS beamline (BL-09) at INDUS-2 synchrotron source*, AIP Conference Proceedings, 2014; American Institute of Physics: 2014; pp 649-651.**

**3. Petschke, D.; Staab, T. E., DDRS4PALS: A software for the acquisition and simulation of lifetime spectra using the DRS4 evaluation board. *SoftwareX* 2019, 10, 100261.**

**4. Olsen, J.; Kirkegaard, P.; Eldrup, M. In *Analysis of positron lifetime spectra using the PALSfit3 program*, AIP Conference Proceedings, 2019; AIP Publishing: 2019.**

**5. Kresse, G.; Furthmüller, J., Efficient iterative schemes for ab initio total-energy calculations using a plane-wave basis set. *Physical review b* 1996, 54, (16), 11169.**

**6. Kresse, G.; Joubert, D., From ultrasoft pseudopotentials to the projector augmented-wave method. *Physical review b* 1999, 59, (3), 1758.**

**7. Blöchl, P. E., Projector augmented-wave method. *Physical review b* 1994, 50, (24), 17953.**

**8. Perdew, J. P.; Burke, K.; Ernzerhof, M., Generalized gradient approximation made simple. *Physical review letters* 1996, 77, (18), 3865.**

**9. Monkhorst, H. J.; Pack, J. D., Special points for Brillouin-zone integrations. *Physical review b* 1976, 13, (12), 5188.**

**10. Paier, J.; Marsman, M.; Hummer, K.; Kresse, G.; Gerber, I. C.; Ángyán, J. G., Screened hybrid density functionals applied to solids. *The Journal of chemical physics* 2006, 124, (15).**
